# ‘Isotopo’ a database application for facile analysis and management of mass isotopomer data

**DOI:** 10.1093/database/bau077

**Published:** 2014-09-08

**Authors:** Zeeshan Ahmed, Saman Zeeshan, Claudia Huber, Michael Hensel, Dietmar Schomburg, Richard Münch, Eva Eylert, Wolfgang Eisenreich, Thomas Dandekar

**Affiliations:** ^1^Department of Bioinformatics, Biocenter, University of Würzburg, Am Hubland, 97074 Wuerzburg, Germany, ^2^Department of Neurobiology and Genetics, Biocenter, University of Wuerzburg, Am Hubland, 97074 Wuerzburg, Germany, ^3^Institute of Molecular and Translational Therapeutic Strategies, OE 8886, Hannover Medical School, Carl-Neuberg-Str. 1, D-30625 Hanover, Germany, ^4^Lehrstuhl für Biochemie, Center of Isotopologue Profiling, Lichtenbergstraße 4, Technische Universität München, D-85747 Garching, Germany, ^5^Division of Microbiology, Barbarastraße 11, Gebäude 36, University of Osnabrück, 49076 Osnabrück, Germany, ^6^Department of Bioinformatics and Biochemistry, Langer Kamp 19B, Technical University Braunschweig, D-38106 Braunschweig, Germany, ^7^Institute for Microbiology, Biozentrum, 2. Obergeschoss Spielmannstraße 7, Technical University Braunschweig, 38106 Braunschweig, Germany and ^8^Computational biology and structures program, European Molecular Biology Laboratory, Meyerhofstr. 1, 69117 Heidelberg, Germany

## Abstract

The composition of stable-isotope labelled isotopologues/isotopomers in metabolic products can be measured by mass spectrometry and supports the analysis of pathways and fluxes. As a prerequisite, the original mass spectra have to be processed, managed and stored to rapidly calculate, analyse and compare isotopomer enrichments to study, for instance, bacterial metabolism in infection. For such applications, we provide here the database application ‘Isotopo’. This software package includes (i) a database to store and process isotopomer data, (ii) a parser to upload and translate different data formats for such data and (iii) an improved application to process and convert signal intensities from mass spectra of ^13^C-labelled metabolites such as tertbutyldimethylsilyl-derivatives of amino acids. Relative mass intensities and isotopomer distributions are calculated applying a partial least square method with iterative refinement for high precision data. The data output includes formats such as graphs for overall enrichments in amino acids. The package is user-friendly for easy and robust data management of multiple experiments.

**Availability: **The ‘Isotopo’ software is available at the following web link (section Download): http://spp1316.uni-wuerzburg.de/bioinformatics/isotopo/. The package contains three additional files: software executable setup (installer), one data set file (discussed in this article) and one excel file (which can be used to convert data from excel to ‘.iso’ format). The ‘Isotopo’ software is compatible only with the Microsoft Windows operating system.

**Database URL:** http://spp1316.uni-wuerzburg.de/bioinformatics/isotopo/.

## Introduction

Incorporation experiments using simple stable-isotope labelled precursors can result in unique isotopologue patterns of metabolic products reflecting the biosynthetic history of the metabolite under study. On the basis of multiple metabolite analysis, isotopologue patterns can be used to reconstruct metabolic pathways and interactions even under complex experimental conditions (1). For simple settings, such as bacteria grown under chemostat conditions with a single carbon source, it is possible to derive accurate metabolic flux distributions from such data (2–4). In practice, the computational analysis of such isotopologue data is still a challenge (5–8). Extending previous efforts in mass isotopomer distribution analysis [MIDA; (1–8)], we first improved our own algorithm and software interface for mass spectrometry (MS) data (9) by including a partial least square calculation. An important challenge in isotopologue profiling is that metabolite data have to be collected on a large-scale to provide the required data basis for the interpretation of the underlying metabolic networks. Therefore, we have created a database manager that enables rapid conversion and storage of multiple isotopomer data. This includes a specific parser that allows data exchange between different formats as well as for direct reading of different data formats into ‘Isotopo’. Moreover, the output formats have been extended, enabling data comparison on the basis of graphs displaying the ^13^C-profiles in compound families such as amino acids.

As a result, the database application ‘Isotopo’ has been established including (i) a large-scale database to store and process multiple isotopologue data, (ii) a useful parser to upload and translate different data formats for such data and (iii) an improved application to directly process MS spectra of ^13^C-labelled compounds such as tertbutyldimethylsilyl (TBDMS)-derivatives of amino acids. Relative mass intensities are herein calculated applying a partial least square method and, for optimal resolution of the isotopologue data, iterative refinement is effected. The freely available package includes a tutorial and allows robust data management of multiple labelling experiments.

## Motivation and results

**Figure 1.**
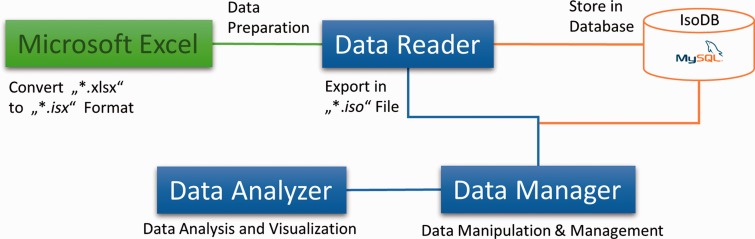
*Components of the Isotopo*: This figure is the abstract, visual presentation of the workflow of the components (Microsoft Excel Data file, Data Reader, Database, Data Manager and Data Analyser) of the *Isotopo*.

**Figure 2.**
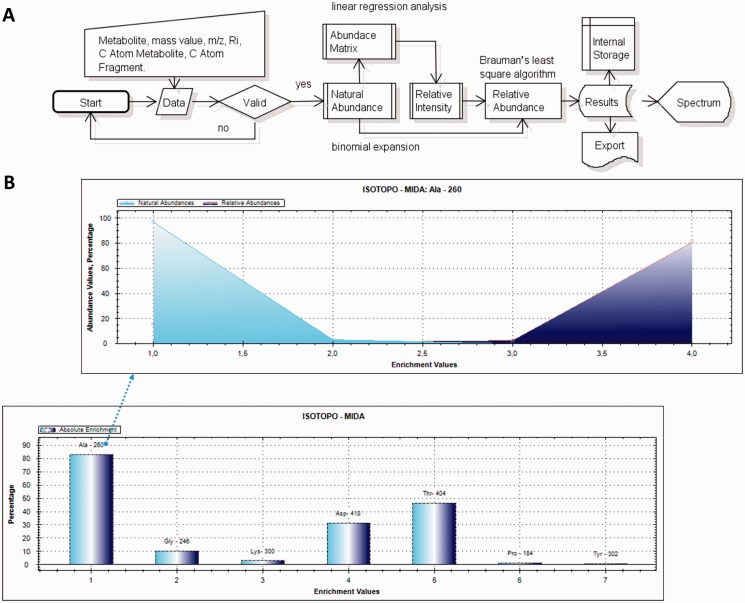
*Work flow of Isotopo and presentation of results:* (**A**) Visual presentation of the unified mark-up language- based flow chart. The implemented flow of operations performed during experimental data input, processing, analysis and visualization is given. The presented workflow starts with the input of raw data, which are first validated and then analysed to estimate the natural abundance values (using abundance matrix and binomial expansion), relative intensity values and relative abundance values (using Brauman’s least square algorithm). Finally, the analysed results are visualized as a bar plot, stored in the internal storage and available to export. (**B**) The bar plot is based on the estimated absolute enrichment values of various amino acids from a labelling experiment with *Salmonella typhimurium*: alanine-260, glycine-246, lysine-300, aspartic acid-418, threonine-404, proline-184 and tyrosine-302, whereas the mountain plot gives in the example a detailed view based on the calculated natural and relative abundance values of alanine-260. Bar and mountain plots are different options to visualize different data sets.

Different data sets were tested (metabolites from different bacterial strains and media). To apply Isotopo, experimental data have first to be collected from gas chromatography– mass spectrometry (GC-MS) experiments. The experimental process using Isotopo consists of three major steps: data preparation, analysis and visualization as shown in Figure 1, following the implemented workflow as shown in Figure 2a.

Example results and graphics are shown in Figure 2b. Representation includes mountain plot and bar plot, so that different spectra based on the absolute enrichment values, and abundances against mass-to-charge ratios. The example is based on the computational analysis of seven different TBDMS-derivatives of amino acids (alanine-260, the fragment number is 260, adding one molecule alanine, two molecules TMS (trimethylsilyl) and subtracting one methyl group, the fragments are thus numbered according to their total molecular weight, fragment charge z = 1; accordingly, fragment numbers are given for glycine-246, lysine-300, aspartic acid-418, threonine-404, proline-184 and tyrosin-302; format: amino acid-fragment number) from labelling experiments with *Salmonella* (details in Supplementary Material). Based on the obtained results, a clear difference is observed in actual (experimental data) and calculated (estimated) natural and relative abundance values. The database applications were successfully used in various experiments on isotopologue data to study the metabolism of bacteria under different growth conditions.

Before we started our database application effort, data were scattered in different formats, no database was available and results were calculated by in-house software using Excel macros or resorting to more limited academic or commercial solutions. For instance, the program Envelope is a visualization software package for already calculated isotope distributions. With a user-friendly interface, the displayed isotope distributions change in near real time in response to user-controlled changes in the labelling parameters using continuously variable slider controls or text input boxes (10). The Theoretical Isotope Generator is an application developed to gain isotopologue-related information on molecules and their relative intensity for educational purposes (chemists, students, lecturers and researchers) (11). For comparison, METATOOL, one of a number of related tools for modelling purposes, allows calculation of fluxes in various metabolic networks relying on biochemical reactions, external and internal metabolites but requiring and relying on no further experimental data (12). The software Isodyn (13) simulates the dynamics of isotopic isomer distribution in central metabolic pathways, and is further enhanced by algorithms facilitating the transition between various analysed metabolic schemes as well as tools for model discrimination. With the software least square mass isotopomers distribution analysis (LS-MIDA) (9), we studied an implementation of a MIDA calculation software; however, the refined Isotopo database application presented here provides, together with the database and its management, an improved MIDA calculation algorithm (rectangular matrix, partial least square calculation) with iterative refinement for automatic, direct and everyday isotopologue data analysis and management. The Isotopo database application now delivers full control over the data, comparison between data sets as well as data management. All these options are important enhancements for long-term and accurate usage in large-scale isotopologue analyses and experiments.

By these and similar experiments on *Salmonella* and *Listeria*, a detailed analysis of amino acid metabolism and its connection to carbohydrate metabolism in infections was possible (14, 15). In general, insights provided by the new software on bacterial metabolism (e.g. *Staphylococcus aureus*) include better understanding of metabolic changes and adaptation, alternative processing routes and energy and yield considerations.

Specifically, we wanted to investigate how *Salmonella* obtain nutrients and metabolites in the *Salmonella*-containing vacuole. Alternative hypotheses envision a direct transport and redirection of nutrients from the *Salmonella* cytoplasm into the vacuole or stress the autotrophic potential of the *Salmonella*, so that only a basic C- or N-source is required. To get an insight into this, numerous data sets had to be assembled applying our application. Current data indicate, for a number of amino acids (e.g. glutamate, aspartate), that rapid and direct synthesis is possible. Additionally, a clear indication for transport processes (e.g. glucose transporters) could be obtained. The database application has now been made open for the community, so that of course completely different biological questions and experiments can be efficiently stored and analysed through exploiting isotopologue data.

## Methodology

The Isotopo is developed using the Microsoft C# (sharp) programming language and Microsoft Dot Net framework, which is why it is compatible only with the Microsoft operating systems. The Isotopo and all its provided material are available for free usage, and by downloading and using it, users agree to these license conditions.

### Data analyser

**Figure 3.**
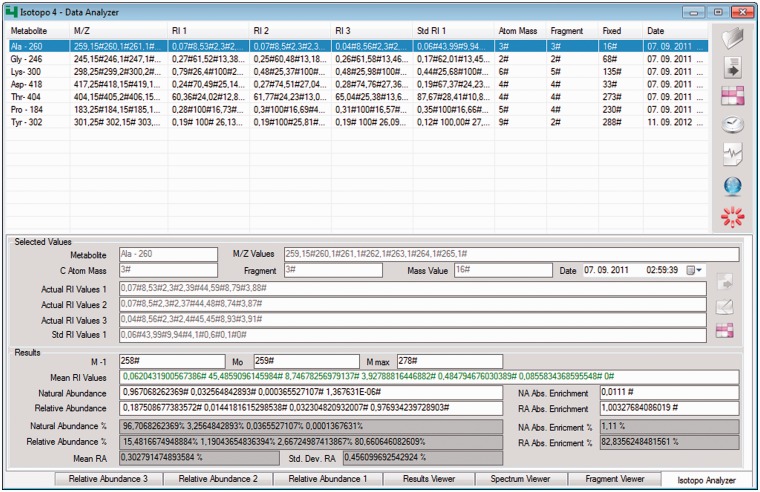
*Isotopo data analyser*: GUI. The top right part of the GUI contains the options to open the file, remove loaded data (if it exists), measure the results, perform measurements of the whole data set, delete specific data, connect to the database to load data and close the application. The white list in the GUI (middle, left) is the container and holds the data. The bottom part of the GUI presents the estimated results and allows editing of all selected data. Data from three experimental data sets can be compared (relative abundance data sets 2 and 3 are in the back and not selected).

This is the most important (backbone) module of the application, which provides options for the experimental data load, analysis and visualization (Figure 3). It allows the user to enter experimental data manually or to load data from existing files and to connect the application to the database server (if available) to fetch data. We have introduced new data standards for efficient data extraction and effective data management, which speed up preprocessed data processing operations. We have standardized data in two different formats: ‘*.iso’ (this is the extension for normal isotope files, which can be used in both data management and data analyser modules) and ‘*.isx’ (this is the extension for data generated by the data reader, after parsing existing data lists, e.g. from Excel files). The major reason for this new standardization of experimental raw data is to categorize the data in personalized formats to fasten the process of data processing. The ‘*.iso’ and ‘*.isx’ formats consist of the following by semicolon ‘;’ separated elements: metabolite name, mass-to-charge ratio values, first, second and third set of relative intensity values (from three independent measurements), standard relative intensity values, atom mass, fragment, fixed value and date and time. Multiple metabolite values are separated by an asterisk ‘*’.

The data analyser now processes the experimental data, individual data elements (one single metabolite information, for example, for the alanine-260 fragment) and data sets with multiple data entries at once (sets of different metabolites, for example, glycine-246, lysine-300, aspartic acid-418). Input data are based on the following information: metabolite name, mass-to-charge ratio values, number of fragments, mass values, number of atoms and actual and standard intensity values. In return after the analysis, it produces the following results: minimum to maximum mass values of a selected fragment (M_-1_, M_0_, M_max_), mean relative intensity values of mass signals, natural abundance values and relative abundance values, which indicate here and throughout the manuscript relative intensities of isotopomers in labelled compounds. Furthermore, it gives absolute enrichments of estimated natural and relative abundance values, so that overall abundances of isotopomers in natural abundance compounds are compared with mean values and standard deviation of experimentally measured (three times) abundance values.

Resulting values are presented in six different subsections of the data analyser: The fragment viewer provides the information about the number of groups with each group’s respective natural and relative abundance values (the latter according to the experimental data; together, they allow an estimate of the absolute enrichment); spectrum viewer (provides the visualization of produced results in different modes: bar, curve, filled curve and labels); results viewer provides all resultant data for all sets of experiments. This helps, especially when the user needs to analyse a complete data set consisting of multiple data entries. Furthermore, entered, loaded, edited or analysed results data can be saved in data files and further reused as required.

The detailed methodological, mathematical and feature-based information, along with produced results (using GC-MS preprocessed experimental data), is given in the Supplementary Material.

To meet the aforementioned application’s goals, the graphical user interface (GUI) of Isotopo data analyser consists of nine main controls (open data file, clear all text controls, measure selected data, process all data, remove selected data, open data manager, close Isotopo, select values and results) and seven views (Isotopo Analyser, Fragment Viewer, Spectrum Viewer, Result Viewer, Relative Abundance 1, Relative Abundance 2 and Relative Abundance 3), as explained in the attached Supplementary Material.

### Database manager

One major focus during the development of a new system towards MIDA is to create a system with fewer dependencies, especially in terms of data management, analysis, visualization and sharing. During the software planning, we found that the major issues are not related to the archiving of data (e.g. which database to use), but to the handling of data. In our case, the experimental (GC-MS) raw data typically come in Microsoft Excel sheets, separated into the different formats, which later need to be analysed. Furthermore, the aim was to have a system with a personalized data management module, which can easily work offline as well. Keeping these requirements in mind, we designed and implemented the new system, which provides a third-party-independent personalized file-based data management system. It allows the user to create, edit, load (previously created), delete and update data files with defined extension (‘*.iso’).

One cannot deny the importance of available data management systems (e.g. MySQL, Oracle, PostgreSQL) to archive, access and secure huge amounts of data. Based on our previous experience, we preferred here a MySQL database solution as it provided Isotopo with a flexible and performant solution. To further extend the scope of data management and feature-based development, we have also designed a new database scheme (given in the attached Supplementary Material, Section 7). The data may be kept in an in-house, intranet database, and when desired the system is setup such that only authorized users can connect to the created Isotopo database server (IsoDB) to get and set up the data. It allows the user to perform direct data manipulation and management operations using the created database, which can later be directly accessed by the data analyser module for further processing. However, both options—connection to the Internet (web server option) and direct local storage of the data—are provided, which allow more flexibility than one option alone.

**Figure 4.**
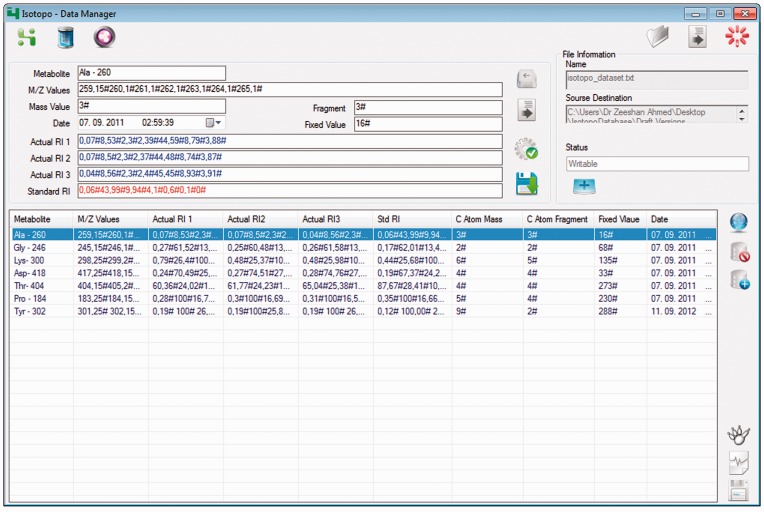
*Isotopo database manager*: GUI. The top right part of the GUI allows the user to load an existing data file. The top left part of the GUI allows the user to enter new, edit selected and delete values shown in the bottom left list view. The bottom right part allows the user to connect to the database, load and delete data from database.

To meet the aforementioned application’s goals, the GUI of Isotopo Data Manager (Figure 4) consists of 16 main controls (open data file, clear all text controls, close Isotopo data manager, add new values, update edited values, clear text fields, save data in file, select values to edit, delete values, create new data file, select source directory, save file, cancel creating file, data view, Open Isotopo Data Analyser and Open Isotopo Data Viewer), as explained in the attached Supplementary Material.

### Data format parser

**Figure 5.**
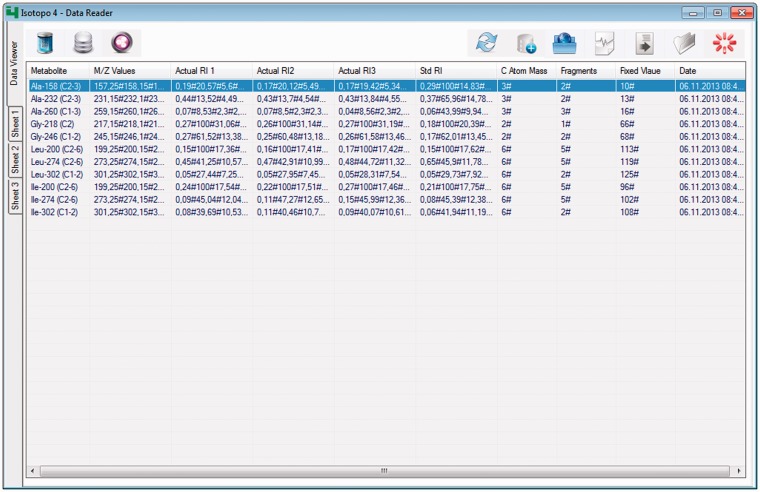
*Isotopo data format parser*: GUI. This is the main interface of the module, which presents the converted data from Excel data files. Moreover, it provides options at the top right to open, refresh or load data into the database, export data into a file, delete data and close the application. The top left options are to browse the data analyser, database manager and data viewer.

This utility module of the Isotopo application (Figure 5) helps the user in transferring data from a different data format (e.g. Microsoft Excel) to the “*.isx” data format. This module is based on a newly programed, dedicated data classifier based on supervised machine learning principles.

The programed data classifier automatically merges all the data from three sheets into a simple and readable format (Figure 3). Preprocessed GC-MS data from Excel data files are rapidly parsed and integrated into the database. The data are divided into three parts: Supplementary Table S1 contains the information about metabolites, mass-to-charge ratio values with actual relative intensity values. Supplementary Table S2 contains the information about similar metabolites and mass-to-charge ratio values including standard relative intensity values. Supplementary Table S3 contains the essential information about the number of fragments, atoms, mass values and groups. An example Microsoft Excel data file is provided in Supplementary Material (‘*.xlsx’ are used in 64-bit operating systems with Microsoft Office version greater than 2003, and ‘*.xls’ are used in 32-bit operating systems with Microsoft Office version 2003 or older).

Our developed data classifier intelligently reads all tables and transfers the data into our software (see Supplementary Material for detailed examples).

The user can not only read and validate the data using Excel data files but also edit them using the Isotopo data format parser. The user can also manually convert the data into the ‘*.isx’ format. Later, the user can export converted data into the data files (‘*.isx’) as well as directly load them to the connected database server, which can be easily accessed by the database manager for further editing, etc., and data analyser for further analysis [just follow the format of data given in the provided Excel sheet (attached in Supplementary Material)].

### Raw data processing by the Isotopo software

Different calculation algorithms for MIDA have already been given (4–6, 16–18). MIDA has been quantitatively validated and compared by independent methods, including a biosynthesis polymer measurement (1, 2, 7, 8). A binomial expansion is used for the measurement of natural abundance values. An abundance matrix is drawn and multiple regression analysis is performed. As a specific algorithmic improvement to previous software including our own (9), we introduce an improved partial least square calculation and algorithm for the measurement of relative intensity values with respect to each *m/z* values. Using estimated relative intensity values, there is a newly drawn abundance matrix and pseudo-inverse matrix calculated: we have estimated actual values and percentages of relative abundances, fractional molar abundances and minimum values with respect to the number of fragments (see Supplementary Material for details). This whole procedure is repeated twice to obtain precise values. A third (optional) iteration validates results and convergence. Using resultant natural, relative and fractional molar abundances, absolute ^13^C enrichments, mean and standard deviations are measured.

The reliability in the results produced by this technique and the Isotopo application depends on a number of factors. At first, the analysis is based on the assumption that the fragmentation patterns for all heteroatom isotopes are identical (i.e. no differential isotope effect), the relative abundance (actual) values of the isotopes are known and either the natural abundances are known or measured in some way. For optimal results, pre-filtering by the vendor software in the instrumentation to identify peaks, filtering out solvent contamination and enhancing the signal together with the new Isotopo software for further processing and calculations, is critical, as tested in various metabolic pathway analyses using different pathogens. A tutorial in the Supplementary Material shows that Isotopo is easily used and handled.

Isotopo is capable of analysing three actual relative intensity values together with the use of standard relative intensity values against mass-to-charge ratio values, and, in return, not only estimates the abundances for each used actual intensity value but also the averages of relative and natural abundance. Importantly, besides the improved algorithm for isotopologue value calculation, Isotopo is a database application with the inclusion of the new database management system, data format parser and integrated product line architecture, whereas in all aforementioned tools there is no database management system and utility to automatically convert the data from other formats to the processable data format.

## Conclusion

Isotopologue distributions from MS measurements are rapidly translated into a quantitative pathway analysis applying the open-source and well-designed database application ‘Isotopo’. The software facilitates the usage of MS-based labelling experiments for a broad range of potential users interested in the metabolism of bacteria, host cells and other organisms.

## Supplementary Data

Supplementary Data are available at *Database* Online.

Supplementary Data
