# Supplementary material for: ‘Isotopo’ a database application for facile analysis and management of mass isotopomer data
Source: Database (Oxford). 2014 Sep 8;2014:bau077. doi: 10.1093/database/bau077 (PMC4158277; doi:10.1093/database/bau077)
Supplement: Supplementary Data [file supp_2014_bau077_index.html]

‘Isotopo’ a database application for facile analysis and management of mass isotopomer data — Supplementary Data 

# ‘Isotopo’ a database application for facile analysis and management of mass isotopomer data

## Supplementary Data

files

**Files in this Data Supplement:**

- Supplementary Data - docx file
