# Supplementary material for: ‘Isotopo’ a database application for facile analysis and management of mass isotopomer data
Source: Database (Oxford). 2014 Sep 8;2014:bau077. doi: 10.1093/database/bau077 (PMC4158277; doi:10.1093/database/bau077)
Supplement: Supplementary Data [file supp_bau077_IsotopoSupplement_RevisionF.docx]

**“Isotopo” a Database Application for Facile Analysis and Management of Mass Isotopomer Data**

**Zeeshan Ahmed^1,7^, Saman Zeeshan^1,8^, Claudia Huber^5^, Michael Hensel^2^, Dietmar Schomburg^3^, Richard Münch^4^, Eva Eylert^5^, Wolfgang Eisenreich^5^ and Thomas Dandekar^1, 6,*^**

^1^Department of Bioinformatics, Biocenter, University of Würzburg Germany,

^2^Division of Microbiology, University of Osnabrück Germany,

^3^Department of Bioinformatics and Biochemistry, Technical University Braunschweig Germany,

^4^Institute for Microbiology, Technical University Braunschweig Germany,

^5^Lehrstuhl für Biochemie, Center of Isotopologue Profiling, Technische Universität München Germany,

^6^EMBL, computational biology and structures program, Heidelberg

^7^Department of Neurobiology and Genetics, Biocenter, University of Wuerzburg Germany,

^8^Institute of Molecular and Translational Therapeutic Strategies, Hannover Medical School, Hanover, Germany

^*^corresponding author

**E-mail addresses:**

ZA: [zeeshan.ahmed@uni-wuerzburg.de](mailto:zeeshan.ahmed@uni-wuerzburg.de)

SZ: [saman.majeed@uni-wuerzburg.de](mailto:saman.majeed@uni-wuerzburg.de)

CH: [claudia.huber@mytum.de](mailto:claudia.huber@mytum.de)

MH: [michael.hensel@biologie.uni-osnabrueck.de](mailto:michael.hensel@biologie.uni-osnabrueck.de)

DS: [d.schomburg@tu-bs.de](mailto:d.schomburg@tu-bs.de)

RM: [r.muench@tu-bs.de](mailto:r.muench@tu-bs.de)

EE: [eylert.eva@ch.tum.de](mailto:eylert.eva@ch.tum.de)

WE: [wolfgang.eisenreich@mytum.de](mailto:wolfgang.eisenreich@mytum.de)

TD: [dandekar@biozentrum.uni-wuerzburg.de](mailto:dandekar@biozentrum.uni-wuerzburg.de)

# Supplementary material - additional information on calculation steps and Mass isotopomer Data ANALYSIS (MIDA)

# Isotopo DATA ANALYZER

The data analyzer is capable of easily processing experimental data. Input includes: metabolite information (ion), mass to charge ratio (m/z) values, actual relative mass intensities (up to three entries for a given m/z value from technical replicate measurements), respective relative intensities from reference compounds, and the number of carbon atoms in fragments. During gas chromatography – mass spectrometry (GC-MS) experiments, the m/z values are dimensionless quantities formed by dividing the mass number of the ion by its charge number, the actual relative intensities are the different intensity values for individual ions measured. “Natural abundance” denotes a theoretical value calculated by Isotopo from the natural isotope abundances of atoms which is the complete population of isotopomers in the molecules of a given compound (including all labelled isotopomers), “relative abundance” is a vector calculated that refers to the population of labeled isotopomers (e.g. by 13C) in the molecules of a compound. Relative intensities per m/z value are calculated from m/z values next. Here, the “fractional molar abundance” means the concentration of a molecular species as a fraction in the total amount of molecules (Lee et al., 1990).

The software estimates mass values (M_0_, M_-1_, M_max_): These are three values estimated from m/z values, M_0_ is the first m/z value, M_-1_ is the first m/z value minus 1 and M_max_ is estimated maximum m/z value for an optimal mass window of detection (peaks between M_-1_ and M_max_). For iterative refinement, Isotopo calculates the best estimate, subtracting relative intensity values from the fractional molar abundances. Finally, the absolute enrichment of natural abundances and the absolute enrichment of experimental measured abundances are calculated. Using the implemented application up to three observed relative intensity values for a given m/z value can be efficiently processed. Corresponding mean and standard deviations are calculated, the results are drawn as a curve of calculated relative intensity values. To standardize and maintain the experimental metabolite data, a file-based data manipulation and management system is also implemented independent of third party tools. This new data management system allows the user to create new experimental data-based files as well as to merge new data files to existing files and to manipulate file data in various ways.

The motivation for our research and new software application is to obtain a comprehensive easy-to-use software application, including different calculation options, iterative refinement, a free software for any user, with a data management system and all options as a key step to provide a data base for the subsequent study of metabolic fluxes such as the intertwined metabolism of host and microbial pathogen.

# MIDA Methodology

Here we explain some general considerations on mass isotopologue data analysis (MIDA) which did lead us to our specific, improved calculation of isotopologue data.

*Natural Abundance Value Calculation*

When the natural (theoretical) abundance values are not available (or estimated), the first step towards mass isotopomers distribution analysis is to determine the natural abundance values because these abundances will then be used for the construction of the abundance matrix for multiple regression analysis to estimate contribution of isotopes from tracers to mass spectrum. Natural abundance values can be estimated by the isotope contents of biosynthesized subunits (such as amino acids) from polymerized product (such as proteins) [2]. For this, we used the binomial expansion for the measurement of natural abundances [1]


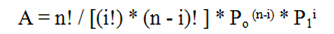


(eq. 1)

Here ‘A’ represents the calculated natural relative abundance values, ‘n’ is equal to the number of atoms,‘i’ is index running from 0 till n-1 and ‘P’ is the distributed proportion of mass isotopomers.

*Abundance Matrix*

To predict the relative isotopomer distribution, at first linear regression analysis is performed using computed natural abundance values by binomial expression (eq. 1).


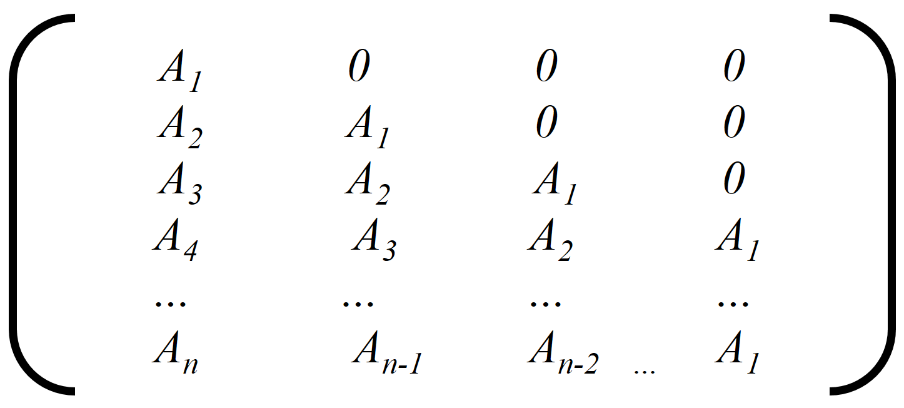


(eq. 2)

Where in eq.2, A_n_, A_n+1_, A_n+2_ … An, are estimated natural abundance values plotted in the form of an Abundance Matrix. The population of this (first) abundance matrix depends upon the number of m/z values e.g. if the number of m/z values is five, then a square matrix of five rows and five columns will be drawn.

*Relative Isotopic Abundance Value Calculation*

Brauman’s algorithm [3] is a least squares technique to calculate relative isotopic abundances (also called as calculated relative intensity values) by simplifying the mass spectra of molecules containing elements with many isotopes by dealing with complex spectra based on fragmentation of molecules. The method has been divided into two parts: first the generation of an appropriate set of linear simultaneous equations and second the solution of these equations. The complete Brauman’s least square algorithm is presented in eq. 3


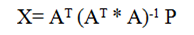


(eq. 3)

But we are using the partial Brauman’s least square algorithm (equation) to calculate relative intensity values, presented in eq. 4.


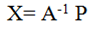


(eq. 4)

X is equal to the product of A inverse times P. Where ‘X’ is equal to the calculated relative intensity values, ‘A’ is the drawn (square) abundance matrix of estimated natural abundance values and ‘P’ is the set of actual relative intensities as observed during a GC-MS experiment.

To compute relative intensity values, linear regression analysis is performed using drawn abundance matrix (eq. 2), with the partial implementation of Brauman’s least square method (eq. 4).


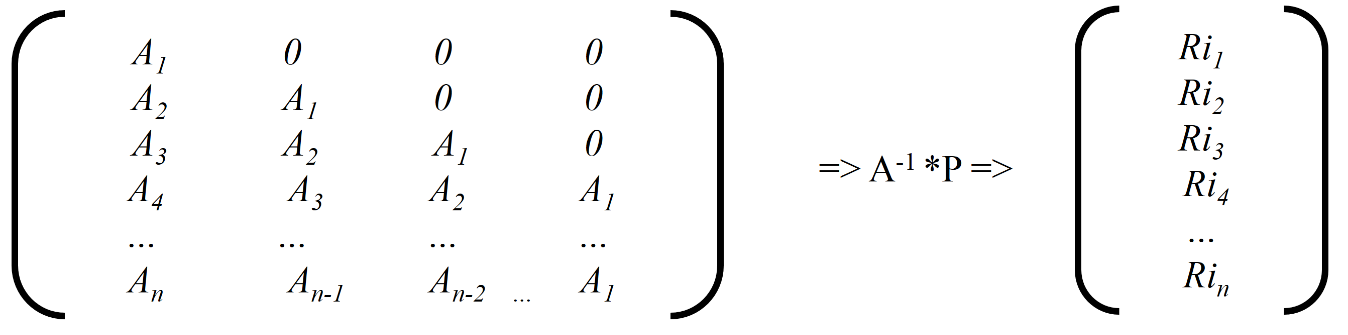


(eq. 5)

As presented in eq. 5, the length of the drawn abundance matrix depends upon the total number of mass to charge ratio and actual relative intensity values. In eq.4, Ri_1_, Ri_2_, Ri_3_, Ri_4_ … Ri_n_, are estimated relative intensity values, estimated with respect to the each m/z values. ‘n’ in this case is the number of total number of m/z values and length of Abundance Matrix, and ‘P’ is the actual observed relative intensity values.

*Relative Abundance Value Calculation*


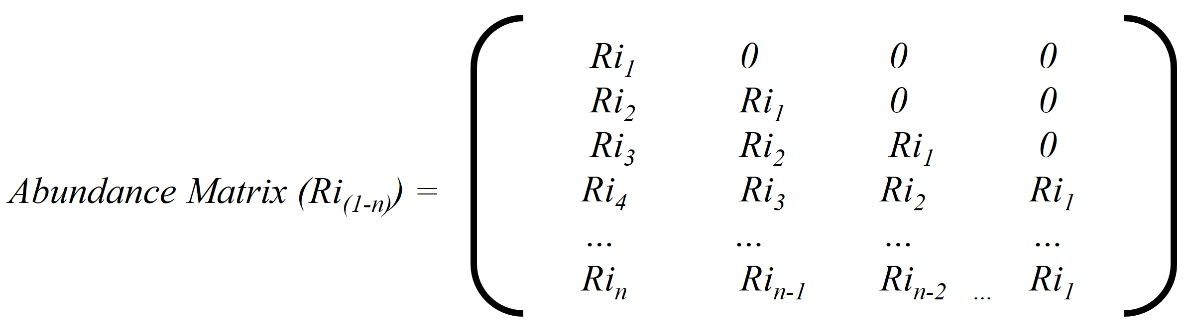


(eq. 6)

In eq.6, R_a1_, R_a2_, R_a3_ … R_an_, are estimated relative abundance values, estimated with respect to the fragment number. “n” in this case is the number of total number of fragments values and the length of a newly drawn (second) Abundance Matrix, and “P” is the observed (standard) relative intensity values.

In most of the cases the number of fragments and relative abundance values will not be equal (in number). This problem is tackled in our new Isotopo software as follows: instead of a square abundance matrix a non-square abundance matrix will be drawn. Mathematical it is not possible to implement eq. 4 as a non-square matrix because the inverse of a matrix can only be computed if it will be a square matrix (number of rows must be equal to the number of columns). To resolve this issue we have used the Pseudo Inverse (also called Generalized Inverse Matrix) of the drawn abundance matrix, as presented in eq. 7.


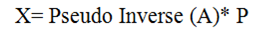


(eq. 7)

X is equal to the product of Pseudo Inverse of A times P. In eq. 7, A is the second drawn abundance matrix consisting of computed relative intensity values and P are the actual observed relative intensity values.

*Fractional Molar Abundance Value Calculation*

Fractional molar abundance is the mass fraction of one element to the total mass of a compound. To calculate fractional molar abundance, the equation is


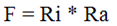


(eq. 8)

F is equal to the calculated relative abundance values times calculated abundance matrix of relative intensity values. Here, ‘Ri’ is the drawn abundance matrix consisting of Ri values (eq. 4), and ‘Ra’ is the measured relative abundance values (eq. 7).

*Minimum Value Calculation*

To calculate minimum values, the equation is


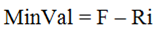


(eq. 9)

MinVal is equal to the calculated fractional molar abundance values minus relative intensity values. There ‘F’ denotes the calculated fractional molar abundance values and ‘Ri’ the respective relative intensity values.

*Mathematical Validation of refinement*

To mathematically validate the calculations, a new linear regression analysis is performed. A new abundance matrix is drawn, whose length is equal to the number of fragments but the difference is that each of its columns consists of new values e.g. if the number of fragments is 2 then three different natural abundance values will be estimated using binomial expansion three times, at first n (in eq. 2) will be set to 3, then to 2 and then to 1, as shown in eq. 10:


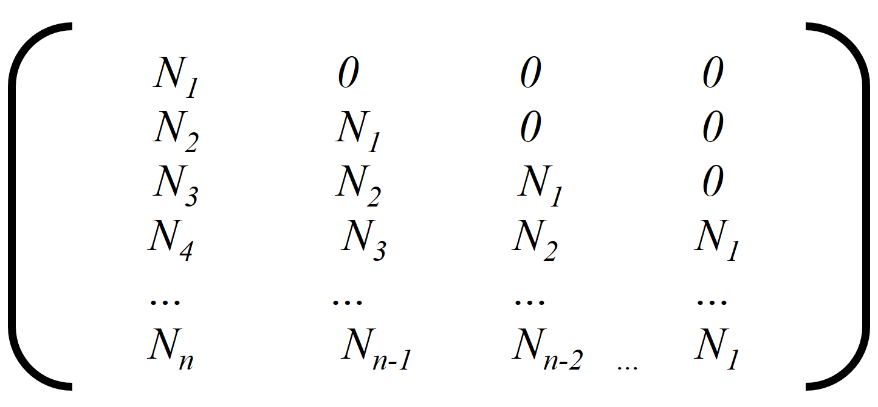


(eq. 10)

N_1_, N_2_, N_3_ … N_n_, are the estimated natural abundance values. Likewise before (eq. 5, 6 and 7), new relative abundance values are estimated, and then the difference is calculated between the newly calculated fractional molar abundance and calculated relative abundance values. A minimum difference validates the result and indicates convergence.

*Absolute Enrichment Estimation*

The proposed equation to compute Absolute ^13^C enrichment of both natural and relative abundances is:


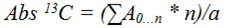


(eq. 11)

Absolute ^13^C Enrichment is equal to the sum of all labelled isotopomers multiplied with their position number (0 to n), divided by the number of carbon atoms of the metabolite) e.g. amino acid) fragment. In eq. 11, A = labelled isotopomer, n = 0 till the number of carbon atoms of the amino acid fragment, and a = number of carbon atoms of the amino acid fragment. Furthermore, to have better analytical measurement view, the percentages of outcomes of these three mathematical equations (natural abundance values, relative abundance values, absolute ^13^C enrichment values and relative intensity values with respect to m/z value) are also computed.

# Technical OveRview

The above mathematics was implemented in the database tool “Isotopo”. The available and tested version of Isotopo database tool provides three main modules i.e. *Isotopo Data Manager, Isotopo Data Analyzer,* as well as *Data format parser* with one additional module i.e. Isotopo Output Viewer.

The *Isotopo Data Manager* is the key utility to store, obtain and manage the multiple data files to be compared in metabolic labelling experiments. It was developed as a user friendly file based experimental data management system. It allows the user to create new experimental data files that later can be used for the analysis using Isotopo Analyzer. It allows the user to perform data manipulation by reading, adding, editing, updating, deleting and merging data (from other source files of the same extension) into a file.

*Isotopo Data Analyzer* is capable of processing experimental data (metabolite information, mass to charge ratio (m/z) values, actual relative intensity values and standard relative intensity values and number of carbon atoms in fragments). It then estimates (definitions see above) mass calculations (Mo, M-1, Mmaximum), predicts natural abundance values, relative abundance values and calculates fractional molar abundance values, the percentage of relative abundance per m/z value and the minimal value. It also draws the spectrum of the calculated relative abundance values. Isotopo Analyzer repeats this whole procedure twice to get new relative abundance values (up to the threshold level of two) because according to the first rule of MIDA, during combinatorial polymer analysis at least two repeats of a probabilistically identical subunit must be present.

The *Data format parser* allows transferring the data from different data environments and formats (e.g. Microsoft Excel) to the “*.isx” data format (is however already explained in detail in the paper).

# Tutorial

To meet aforementioned goals of Isotopo development, the graphical user interface of this application is divided into two main modules i.e. *Data analyzer* and *Data manager* while the *Data format parser* helps to rapidly switch between different data environments and file types.

*Isotopo Data Analyzer* is the module responsible for providing options for experimental data loading, analysis and visualization, whereas *Data Manager* is the module responsible for providing options for experimental data manipulation and management. Isotopo Main is the parent window of the application embedding all the module interfaces and responsible for providing options for child widows manipulation and closing over all application. Finally, Isotopo About is a child interface providing basic information about the current version of in use Isotopo version.

The graphical user interface of Isotopo data analyzer consists of 10 main controls: open data file, clear all text controls, measure selected data, process all data, remove selected data, open data manager, close Isotopo, selected values and results. Moreover, the graphical interface is divided into seven views: Isotopo Analyzer, Fragment Viewer, Spectrum Viewer, Result Viewer, Relative Abundance 1, Relative Abundance 2 and Relative Abundance 3 as shown in Figure 1 (a, b, c, d, e, f, g) and described in Table 1.

The graphical user interface of Isotopo Data Manager consists of 16 main controls: open data file, clear all text controls, close Isotopo data manager, add new values, update edited values, clear text fields, save data in file, select values to edit, delete values, create new data file, select source directory, save file, cancel creating file, data view, Open Isotopo Data Analyzer and Open Isotopo Data Viewer as shown in Figure 2 and described in Table 2.

The graphical user interface of Isotopo Data View consists of seven main controls: Export Data, Remove Selected Values, Clear Values, Import Data, Close Data Viewer, Open Isotopo Data Analyzer and Open Isotopo Data Manager. Moreover the graphical interface is divided into two (tab) views: Result Viewer (Figure 3a) and Fragment Viewer (Figure 3b), explained in Table 3.

# installation

Take the setup executable SBEDA (Software for Biological Experimental Data Analysis) framework and install using Microsoft Windows operating system. The SBEDA framework (including Isotopo) is developed using the Microsoft C# (sharp) programming language and Microsoft Dot Net framework 2008, that’s why it is only compatible to Microsoft operating systems.

For a simple example and guided tour, load the example data into Isotopo Data Analyser by clicking the “Open Data File” icon, and process these by clicking “Process selected data” or “Process all data” icon. Complete software details are provided in Table 3.

# Processing Experimental Data

To apply Isotopo, some experimental data have to be collected first. The process consists of three major steps i.e. preparation of data set, input data file preparation and management, and evaluation and data analysis. Observed data during actual experimentation are collected during the preparation of data set. During input data file preparation and management, at first *Data Manager* is used to structure data by organizing an experimental data file which is later used by *Data Analyzer* for analysis. Additional files are rapidly added using the *Data format parser*. Throughout the experimental data analysis, each observed resultant data during Evaluation is individually analyzed using Data Analyzer and results are obtained.

Observed resultant data during different experiments of metabolic isotopomers analysis with different metabolites are collected for mass isotopomer predictions using Isotopo. The collected example data consist of five different results observed by using six different metabolites during evaluation i.e. Alanine (Ala – 260), Glycine (Gly – 246), Lysine (Lys- 300), Aspartic Acid (Asp - 418), Threonine (Thr- 404), Proline (Pro – 184) and Tyrosin (Tyr – 302) presented in Table 4. The metabolite based data set example presented in Table 4 consists of the following experimental elements i.e. mass to charge ratio (m/z) values, relative intensity values (RI), atomic mass values and atomic fragment numbers.

A data input file for Isotopo Data Analyzer is created using Isotopo Data Manager. During experimental data analysis, the metabolites are taken as input and analyzed using Data Analyzer item by item. The resultant information from the Data analyzer consists of relative intensity per M/Z values, natural abundances, relative abundances per fragments, and absolute ^13^C enrichment, presented in Table 5.

# Isotopo Database

Extending the scope of this research and development towards the proposition of an efficient schematic database structure, this section presents the platform independent relational database schema applied and created using the **Isotopo database tool** (Figure 5 and Table 6)

# Isotopo Designs Modelling

Isotopo implementation follows the principles of a scientific software engineering paradigm (*Butterfly* [4]) to achieve better design, modeling and the development of a scientific software solution. The abstract model of *Butterfly* software design consists of four different phases: scientific software engineering (SSE), human computer interaction (HCI), scientific methodology and scientific application. These four phases help in implementing the proposed methodology in the form of a comprehensive and user friendly prototype application using software engineering principles and human computer interaction guidelines. The software development architecture of the *Butterfly* model consists of three different layers: gray, yellow and green [4].

The principles of the gray layer helped in planning and understanding the processes (mathematics, statistics) to be implemented and creating the logic of the software application by finalizing the functional and non-functional scientific requirements (isotopomers distribution analysis, measurement, visualization and data management) designing the software conceptual models and abstract graphical user interface. Furthermore this layer assisted in the design (mock-ups, abstract designs) and implementation of the graphical interface according to the end user’s background (biochemistry, molecular biology etc.), psychology and working (deployment) environment.

The yellow layer of the *Butterfly* model helped in choosing the best suited design modelling methods including Product Line Architectures (PLA) [5, 6] (set of integrated modules which works independently as well as a single unit software application) UML (Use Case, System Sequence, Activity Flow, Data Flow, Class and Component Diagrams) [7] and database schemas (entity relationship diagrams [8, 9). Furthermore this layer guided the development (efficient preprocessed source code and database script writing) of the system by picking up the most suitable, available, recent, transferable and affordable development tools and technologies, mainly including Microsoft visual studio dot net 2013 Framework, C# object oriented programing language and MySQL enterprise database management system. Please find attached supplementary material for design and technological details.

The green layer of the *Butterfly* model helped with testing operations (black box, white box, integrated, modular) with involvement of the end users, and guides the deployment procedures. Following these *Butterfly* model’s principles, we have implemented the software package “Isotopo”, mainly consisting of three modules (based on product line architecture): Data Analyzer, Database Manager and Data Format Parser. These three modules perform their individual tasks alone and together as a powerful package by their combination.

Isotopo software application is well modelled using UML notations including *Conceptual Design* (Figure 6), *Use Case* (Figure 7), *Data Flow* (Figure 8), *Flow Chart* (Figure 9), *System Sequence* (Figure 10), *Class Diagram* (Figure 11) and *Component Diagram* (Figure 12).

# References

1. Hellerstein, M. K. and Neese, R. A. (1992). Mass isotopomer distribution analysis: a technique for measuring biosynthesis and turnover of polymers. *Am. J. Physiol.*, **263**, 988–1001.
2. Zilversmit, D. B., Entenman, C. and Fishler, M. (1943). On The Calculation of "Turnover Time" and "Turnover Rate" from Experiments Involving the use of Labeling Agents. *J Gen Physiol.*, **26**, 325–331.
3. Brauman, J. I. (1966). Least Squares Analysis and Simplification of Multi-Isotope Mass Spectra. *Anal. Chem.*, **38**, 607–610.
4. Ahmed, Z., Zeeshan, S., Dandekar, T. (2014). Developing sustainable software solutions for bioinformatics using the “Butterfly” paradigm. *F1000Research*.
5. Ahmed, Z. (2010). Towards Performance Measurement and Metrics based Analysis of PLA Applications. *Int. J. Software Engin. App.*, **1**, 66-80.
6. Ahmed, Z. Measurement Analysis and Fault Proneness Indication in Product Line Applications (PLA), In Sixth International Conference on New Software Methodologies, Tools, and Techniques, Italy, 2007, 391-400.
7. Kaur, H. and Singh, P. UML (Unified Modeling Language): Standard Language for Software Architecture Development. In International Symposium on Computing, Communication, and Control, Singapore, 2011.
8. William, K. (1983). A simple guide to five normal forms in relational database theory. *Commun. ACM.*, **26**, 120-125.
9. Fagin, R. (1977). Multivalued dependencies and a new normal form for relational databases. *ACM Trans. on Database Sys.* **2**, 262-278.

Supplementary Material: Tables

**Table 1.** Graphical User Interface Isotopo Data Analyzer; Control Descriptions

| No. | Features | Descriptions |
| --- | --- | --- |
| 1 | open data file | Opens directory browser to select input data file from attached repositories and loads data from data file into data viewer. |
| 2 | clear all text controls | Deletes all loaded data and clears all text controls. |
| 3 | measure selected data | Process selected data entry (only one at a time) from data view and perform MIDA. |
| 4 | process all data | Processes all loaded data (all data entries) at once. |
| 5 | remove selected data | Deletes selected data entry from data view. |
| 6 | open data manager | Opens Isotopo Data Manager’s graphical user interface. |
| 7 | close Isotopo | Closes the Isotopo Data Analyzer. |
| 8 | selected values | Provides text boxes for experimental data manipulation by editing selected input data entry values from data viewer or by entering new experimental data for measurement analysis. It provides following text boxes  Metabolite; name of the metabolite.  M/Z Values; mass to charge ratio values.  C Atom Mass: atom number.  C Atom Fragment; number of fragments  Date  RI 1 Values; relative intensity values.  RI 2 Values; relative intensity values.  RI 3 Values; relative intensity values.  Mass Value; set mass value. |
| 9 | results | Provides text boxes presenting measured results:  M0; mass value  M-1; mass value minus 1  Mmax; maximum mass values  Mean RI Values  Natural Abundance Values  Relative Abundance Values  Percentages of Natural Abundance Values  Percentages of Relative Abundance Values  Absolute Enrichment value of Natural Abundance  Absolute Enrichment value of Relative Abundance  Percentage of Absolute Enrichment value of Natural Abundance  Percentage of Absolute Enrichment value of Relative Abundance  Mean Relative Abundance (calculated from three estimated RAs)  Standard Deviation of Relative Abundance (calculated from three estimated RAs) |
| 10 | Isotopo Analyzer | It is the default view (containing options 1,2,3,4,5,6,7,8,9) |
| 11 | Fragment Viewer | Provides the information about measured abundances (natural and relative) with respect to the number of fragments. It consists of four controls as well  Export File; allows user to export measure fragment based output in a new file.  Import File; allows user to import already estimated data.  Clear Text; allows user to clear the view by deleting all the data.  Delete Selected Data; allows user to delete particular (selected) data. |
| 12 | Spectrum Viewer | Draws a spectrum (visualization) of estimated natural and relative abundance values with respect to the number of fragments. |
| 13 | Result Viewer | Provides the information of complete output including Metabolite name, input values (e.g. M/Z values, Fragment Number etc.) and measured values (e.g. NA, RA and RA/MZ etc.). It consists of four controls as well  Export File; allows user to export measure fragment based output in a new file.  Import File; allows user to import already estimated data.  Clear Text; allows user to clear the view by deleting all the data.  Delete Selected Data; allows user to delete particular (selected) data |
| 14 | Relative Abundance 1 | Provides measured Relative Intensity, Fractional Molar Abundance, Minimum Values using inputted actual RI 1 values. |
| 15 | Relative Abundance 2 | Provides measured Relative Intensity, Fractional Molar Abundance, Minimum Values using inputted actual RI 2 values. |
| 16 | Relative Abundance 3 | Provides measured Relative Intensity, Fractional Molar Abundance, Minimum Values using inputted actual RI 2 values. |

**Table 2.** GUI LS-MIDA Data Manager with control descriptions

| No. | Features | Descriptions |
| --- | --- | --- |
| 1 | open data file | Opens directory browser to select input file from attached repositories and loads data from data file into data viewer. |
| 2 | clear all text controls | Deletes all loaded in data and clears all text controls. |
| 3 | close isotopo data manager | Closes the Isotopo Data Manager. |
| 4 | add new values | Add newly entered values in text boxes to data view. |
| 5 | update edited values | Updates edited values in to data view |
| 6 | clear text fields | Deletes data a from text controls. |
| 7 | save data in file | Saves data into file. |
| 8 | select values to edit | Allows user to select one value from data view to edit existing values. |
| 9 | delete values | Deletes selected data entry from data view. |
| 10 | create new data file | Allows user to create new data (input) file. |
| 11 | select source directory | Opens directory browser to select the directory to store newly created data file. This option is only enabled and visible when user will click option 10. |
| 12 | save file | Allows user to save newly created file. It also allows user to save exiting file data e.g. if some data file is also open and user want to creates a new file, then system will ask if he want to merge existing data (in data view) to newly created file or not. This option is only enabled and visible when user will click option 10. |
| 13 | cancel creating file | Allows user to cancel new file creation process. This option is only enabled and visible when user will click option 10. |
| 14 | data view | Provides the textual view of all loaded, added or updated data. |
| 15 | Open Isotopo Data Analyzer | Opens the graphical user interface of Isotopo Data Analyzer. |
| 16 | Open Isotopo Data Viewer | Opens the graphical user interface of Isotopo Data Viewer. |

**Table 3.** GUI LS-MIDA Data Viewer; Control Descriptions

| No. | Features | Descriptions |
| --- | --- | --- |
| 1 | Export Data | Allows user to export data (into file). |
| 2 | Remove Selected Values | Removes selected data from list view. |
| 3 | Clear Values | Deletes all contained data from all controls. |
| 4 | Import Data | Allows user to import data in to data viewer. |
| 5 | Close Data Viewer | Exits isotopo data viewer |
| 6 | Open Isotopo Data Analyzer | Opens the graphical user interface of Isotopo Data Analyzer. |
| 7 | Open Isotopo Data Manager | Opens the graphical user interface of Isotopo Data Manager. |
| 8 | Result Viewer | Default view, allows user to view, import and export resultant data (obtained from Isotopo Data Analyzer). |
| 9 | Fragment Viewer | Allows user to view, import and export resultant fragment based data obtained from Isotopo Data Analyzer (natural and relative abundance values). |

**Table 4.** Isotopo processing experimental data set

| No. | Metabolitefragment number | M/Z | RI1 | RI 2 | RI 3 | Std. RI | C-atoms per metabolite | C-atoms per fragment |
| --- | --- | --- | --- | --- | --- | --- | --- | --- |
| 1 | Ala 260 | 259,15#260,1#261,1#262,1#263,1#264,1#265,1# | 0,07#8,53#2,3#2,39#44,59#8,79#3,88# | 0,07#8,5#2,3#2,37#44,48#8,74#3,87# | 0,04#8,56#2,3#2,4#45,45#8,93#3,91# | 0,06#43,99#9,94#4,1#0,6#0,1#0# | 3# | 3# |
| 2 | Gly - 246 | 245,15#246,1#247,1#248,1#249,05#250,1# | 0,27#61,52#13,38#11,9#1,91#0,72# | 0,25#60,48#13,18#11,92#1,94#0,73# | 0,26#61,58#13,46#12,15#2,03#0,73# | 0,17#62,01#13,45#5,67#0,77#0,14# | 2# | 2# |
| 3 | Lys- 300 | 298,25#299,2#300,2#301,2#302,15#303,15#304,15#305,15#306,15# | 0,79#26,4#100#27,88#13,3#4,59#1,56#0,53#0,16# | 0,48#25,37#100#28,08#13,61#5,55#1,61#1,23#0,1# | 0,48#25,98#100#27,38#13,3#5,52#1,6#1,21#3# | 0,44#25,68#100#26,94#12,23#3,45#0,89#0,23#0,11# | 6# | 5# |
| 4 | Asp- 418 | 417,25#418,15#419,15#420,15#421,15#422,15#423,1#424,1# | 0,24#70,49#25,14#13,5#52,81#18#7,44#1,66# | 0,27#74,51#27,04#14,32#53,75#17,92#7,62#1,72# | 0,28#74,76#27,36#14,47#56,25#19,02#8,01#1,81# | 0,19#67,37#24,23#11,36#2,71#0,68#0,11#0,01# | 4# | 4# |
| 5 | Thr- 404 | 404,15#405,2#406,15#407,2#408,2#409,2#410,2# | 60,36#24,02#12,85#95,21#31,94#14,67#3,21# | 61,77#24,23#13,02#95,41#32,19#14,66#3,38# | 65,04#25,38#13,67#100#33,72#15,5#3,45# | 87,67#28,41#10,88#2,67#0,41#0,13#0,13# | 4# | 4# |
| 6 | Pro - 184 | 183,25#184,15#185,15#186,15#187,15#188,15#189,1#190,1#191,1#192,1# | 0,28#100#16,73#4,76#0,53#0,09#0,65#0,14#0,07#0,01# | 0,3#100#16,69#4,75#0,53#0,09#0,64#0,15#0,07#0,01# | 0,31#100#16,57#4,72#0,53#0,09#0,64#0,15#0,07#0# | 0,35#100#16,66#4,71#0,51#0#0,59#0,13#0,07#0# | 5# | 4# |
| 7 | Tyr – 302 | 301,25# 302,15# 303,15# 304,15# 305,15# 306,15# | 0,19# 100# 26,13# 10,52# 1,82# 0,77# | 0,19#100#25,81#10,45#1,78#0,75# | 0,19# 100# 26,09# 10,64# 1,86# 0,72# | 0,12# 100,00# 27,07# 10,70# 1,86# 0,58# | 9# | 2# |

**Table 5.** Isotopo Analyzer results^1^

| Metabolite | Mo | M-1 | M  max | NA | RA | NA Abs.  Enrich. | RA Abs.  Enrich | RI Values |
| --- | --- | --- | --- | --- | --- | --- | --- | --- |
| Ala – 260 | 260 | 259 | 278 | 96.7068262369%  3.2564842893%  0.0365527107%  0.001367631% | 15.4816674948884%  1.19043654836394%  2.66724987413867%  80.660646082609% | 1,11 % | 82,8356248481561 % | 0.0620431900567386,  45.4859096145984,  8.74678256979137,  3.92788816446882,  0.484794676030389,  0.0855834368595548,  0 |
| Gly - 246 | 245 | 246 | 315 | 97,792321% 2,195358% 0,012321% | 88,683579025702% 2,05911367510443% 9,25730729919358% | 1,11 % | 10,2868641367458 % | 0,173837780166809# 63,4059842371328# 12,3302015032638# 5,51320965535131# 0,662062317395308# 0,127603147274152# |
| Lys- 300 | 298 | 300 | 438 | 94,5718499425015% 5,30765261584471% 0,119152480606485% 0,00133743809761551% 7,506099142245E-06% 1,6850581551E-08% | 91,3473582476577% 5,7525782120635% 0,716447196993507% 1,40002357173458% 0,145986344901996% 0,637606426648751% | 1,11 % | 3,0315041668212 % | 0,465254724600939# 27,1278460884897# 104,216628801106# 22,6031463530065# 11,5317231993134# 2,97087147483536# 0,759492384229862# 0,196668429158787# 0,104276236597961# |
| Asp- 418 | 417 | 418 | 454 | 95,6333804656704% 4,29378308491836% 0,07229395122246% 0,00054098011836% 1,51807041E-06% | 56,2492278513493% 2,65088727019829% 1,25030185391104% 38,9887524406663% 0,860830583875073% | 1,11 % | 31,3902676588799 % | 0,198675398772716# 70,4371935628838# 22,1736761408553# 10,8298875235577# 2,33033364469966# 0,598107216629968# 0,0863453311822733# 0,00611434246089636# |
| Thr- 404 | 403 | 404 | 681 | 95,6333804656704% 4,29378308491836% 0,07229395122246% 0,00054098011836% 1,51807041E-06% | 35,9468011261879% 4,10037838284991% 2,44234207543641% 53,8140915527946% 3,69638686273118% | 1,11 % | 46,3032211607578 % | 91,6730116337053# 25,5912314443616# 10,1584759875684# 2,31594890866677# 0,316912752867383# 0,119898322596564# 0,130299727680376# |
| Pro - 184 | 183 | 184 | 417 | 95,6333804656704% 4,29378308491836% 0,07229395122246% 0,00054098011836% 1,51807041E-06% | 95,5167944103923% 4,29165722700117% 0,103389092575267% 0,0165325811460895% 0,0716266888851002% | 1,11 % | 1,20863497778259 % | 0,365980997739213# 104,549567403106# 12,726313467226# 4,27463161080234# 0,331150730666402# 0# 0,617480626620422# 0,108223721212432# 0,0678704443906936# 0# |
| Tyr-302 | 301# | 302# | 591# | 97,792321% 2,195358% 0,012321% | 98,6939185067522% 1,16912458603015% 0,136956907217704% | 1,11 % | 0,721519200232779 % | 0,122709021294218# 102,254763027543# 25,3855584023978# 10,3587860476445# 1,6662451514464# 0,554382636791268# |

^1^This table provides information on data from three independent experiments [Relative Abundances (1, 2 and 3) using three Relative Intensity Values (RI1, RI 2 and RI 3)]. The symbol “# “, is the number separator. The implemented Isotopo software application is designed for German and English language. Symbol “,” (comma) is used instead of “.” (dot), so to differentiate (especially in German Systems) values we have standardized “#” (hash) symbol.

**Table 6.** Experimental Data Schema Description.

| No. | Relations | Type | Descriptions |
| --- | --- | --- | --- |
| 1 | Metabolite | One to Many | This relation is to maintain metabolite information consisting of name, number, date, time and constant value. |
| 2 | MassToChargeRatio | One to Many | This relation is to maintain information of mass to charge ratio values. |
| 3 | Metabolite_has_  MassToChargeRatio | Many to Many | This is an intermediate table between Metabolite and Mass to Charge Ratio, to maintain many to many relationships. |
| 4 | RelativeIntensityValues | One to Many | This relation is to maintain information about relative intensity values. |
| 5 | Metabolite_has_  RelativeIntensityValues | Many to Many | This is an intermediate table between Metabolite and Relative Intensity Values, to maintain many to many relationships. |
| 6 | StdRelativeIntesity | One to Many | This relation is to maintain information about standard relative intensity values. |
| 7 | Metabolite_has_  StdRelativeIntesity | Many to Many | This is an intermediate table between Metabolite and Standard Relative Intensity Values, to maintain many to many relationships. |
| 8 | Mass Value | One to Many | This relation is to maintain information about ion mass values. |
| 9 | Fragment | One to Many | This relation is to maintain information about number of fragments. |
| 10 | Metabolite_has_  RelativeAbundance | Many to Many | This is an intermediate table between Metabolite and Relative Abundance Values, to maintain many to many relationships. |
|  | RelativeAbundance | One to Many | This relation is to maintain information about relative abundance values. |
| 12 | RelativeNatural  Abundance_has_  Metabolite | Many to Many | This is an intermediate table between Metabolite and Relative Natural Abundance Values. |
| 13 | RelativeNaturalAbundance | One to Many | This relation is to maintain information about relative natural abundance values. |
| 14 | StandardDeviation | One to Many | This relation is to maintain information about estimated standard deviation values. |
| 15 | Mean | One to Many | This relation is to maintain information about estimated mean values. |
| 16 | AbsoluteEnrichment | One to Many | This relation is to maintain information about estimated absolute enrichment values. |

Supplementary Material: Figures


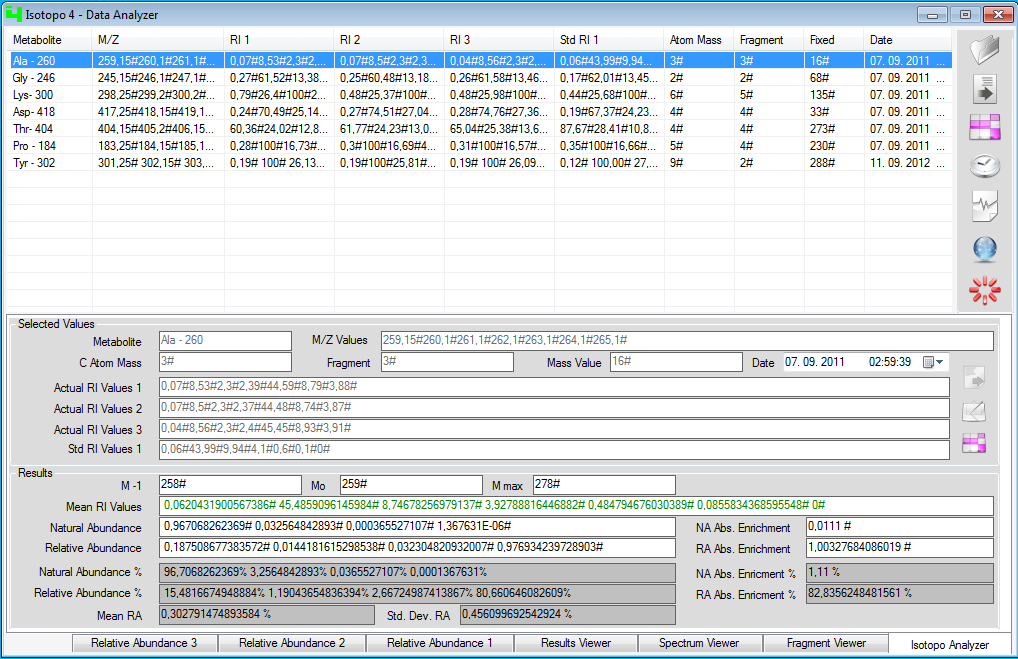


Figure 1a: Isotopo; Data Analyzer Module (Ala-260)


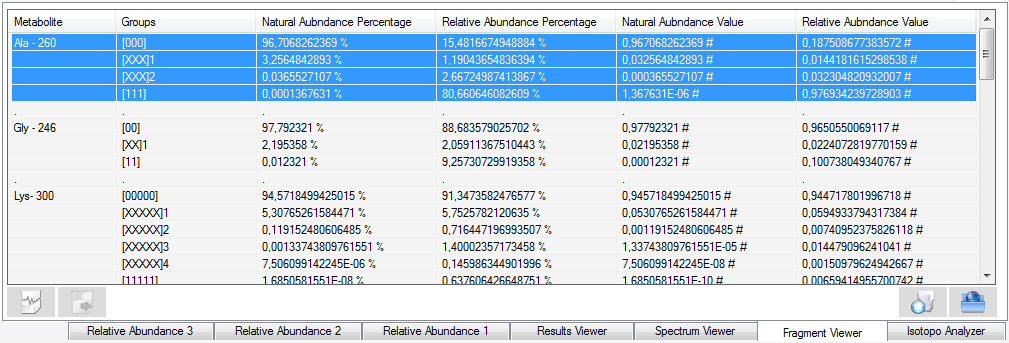


Figure 1b: Isotopo; Fragment Viewer Module (Ala-260)


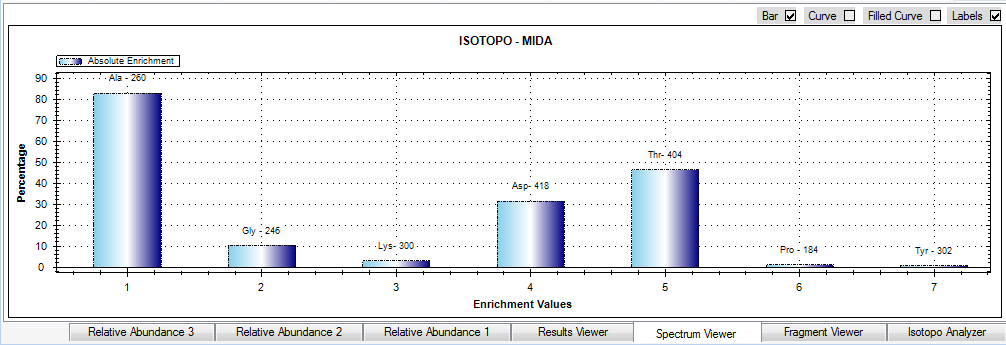


Figure 1c: Isotopo; Spectrum Viewer Module


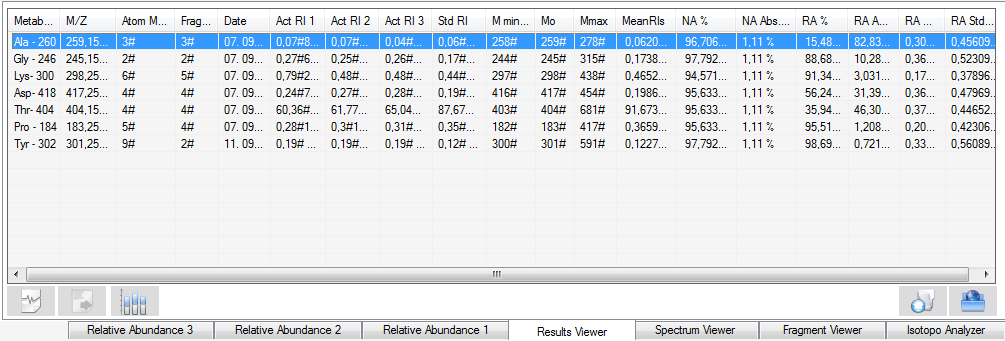


Figure 1d: Isotopo; Result Viewer Module


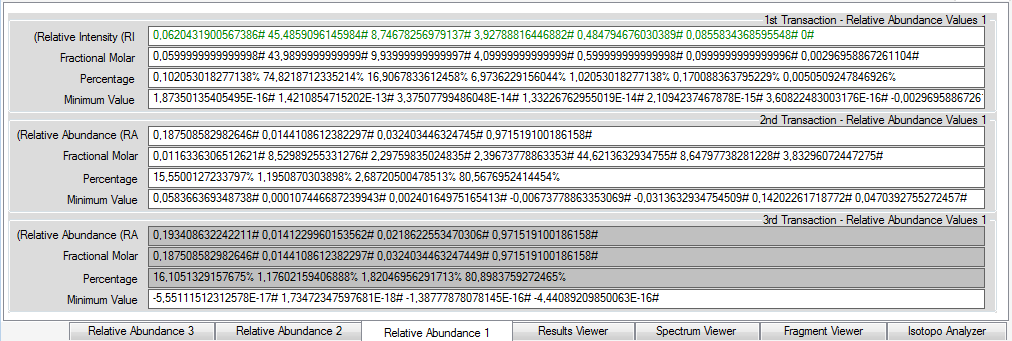


Figure 1e: Isotopo; Relative Abundance 1 Module (Ala-260)


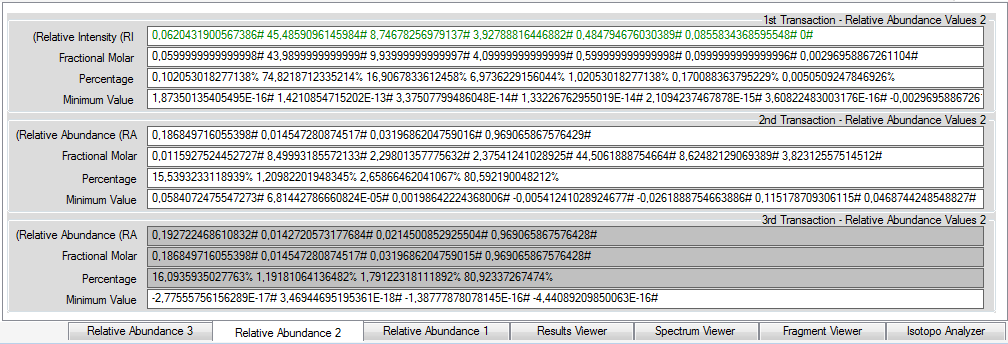


Figure 1f: Isotopo; Relative Abundance 2 Module (Ala-260)


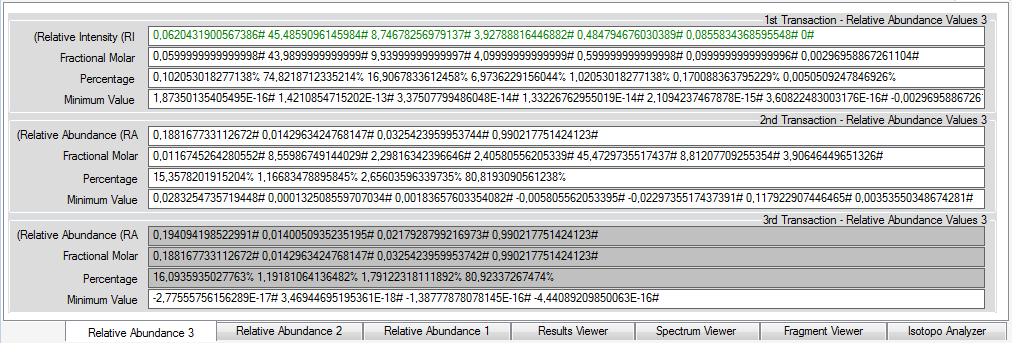


Figure 1g: Isotopo; Relative Abundance 3 Module (Ala-260)

**Figure 1. Isotopo Data Analyzer Graphical User Interface.** (a) Data Analyzer, (b) Fragment Viewer, (c) Spectrum Viewer, (d) Result Viewer, (e) Relative Abundance 1, (f) Relative Abundance 2, (g) Relative Abundance 3.


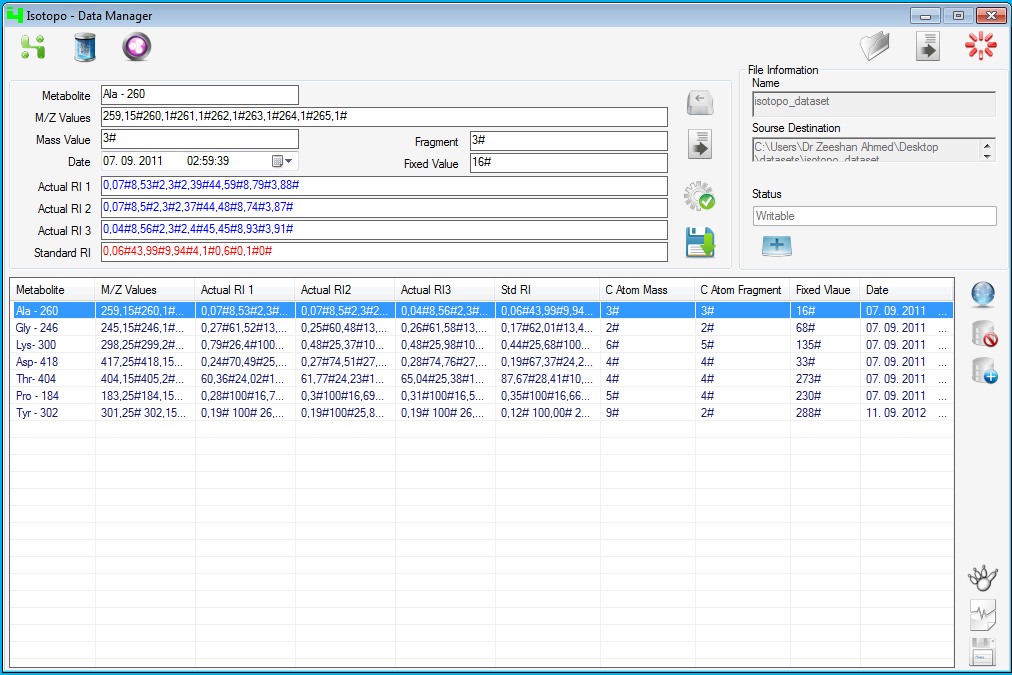


**Figure 2. Isotopo Data Manager Graphical User Interface**


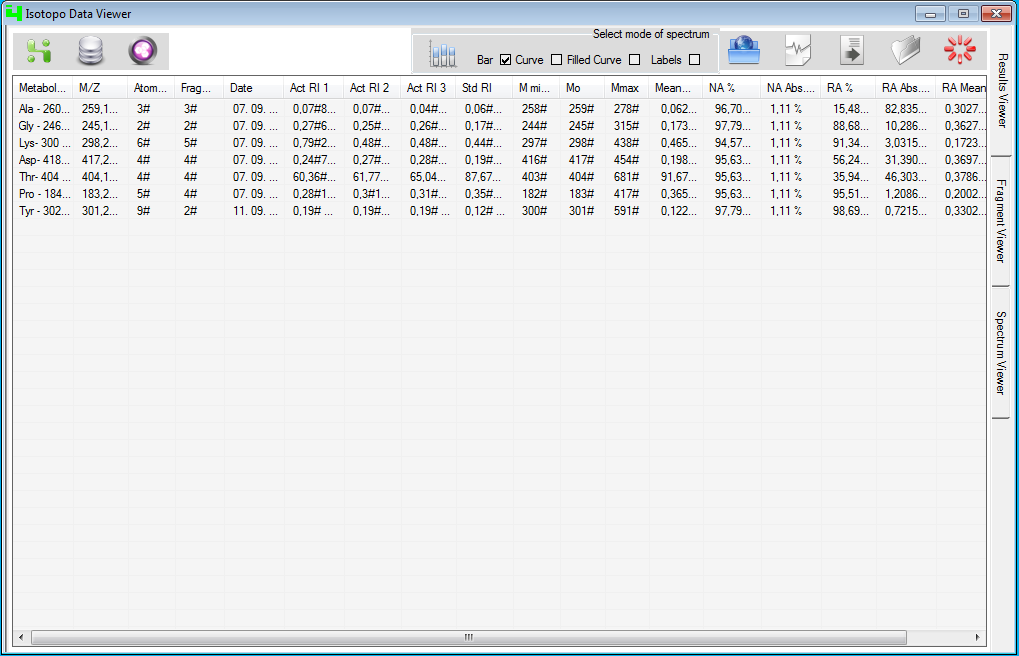


**Figure 3a: GUI of Isotopo Data Viewer (results)**


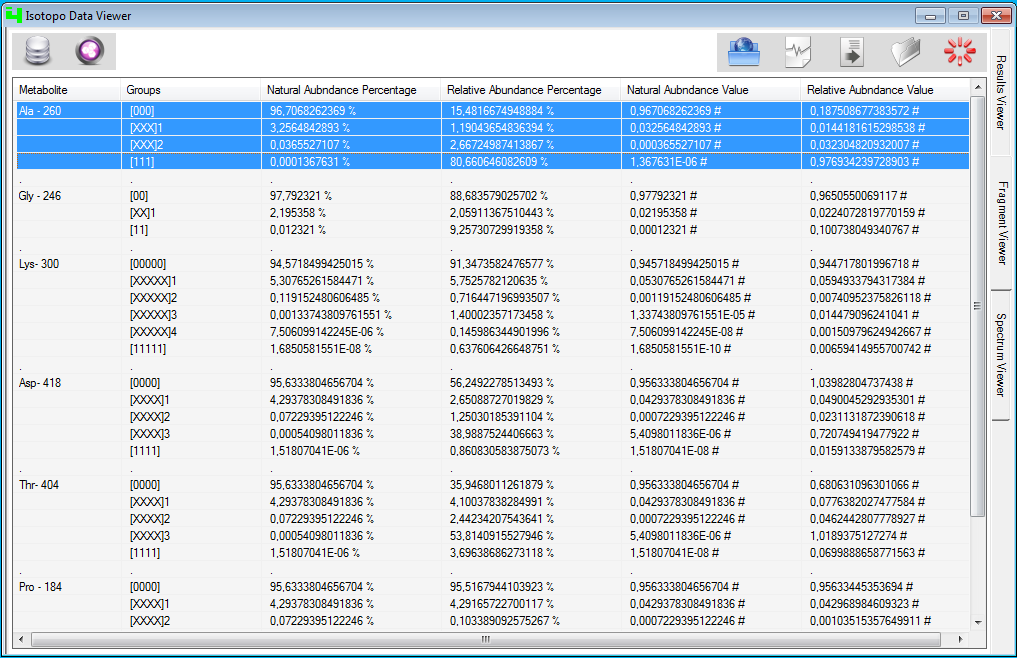


**Figure 3b: GUI of Isotopo Data Viewer (fragments)**


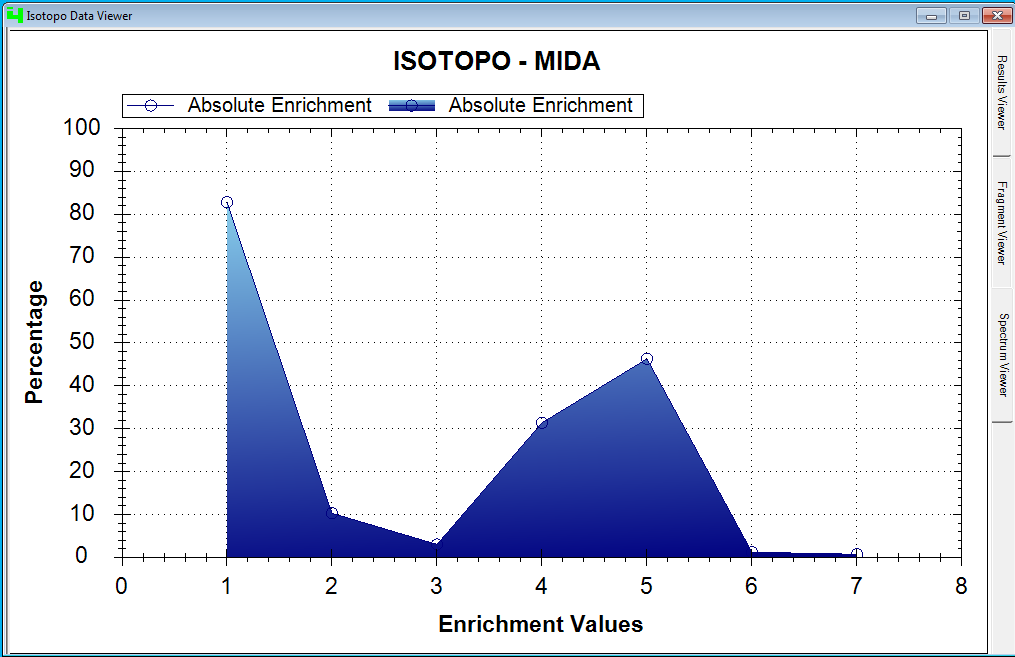


**Figure 3c: GUI of Isotopo Spectrum Viewer (fragments)**

**Figure 3. Graphical User Interface of Data Viewer.** (a) results, (b) fragments and (c) spectrum viewer


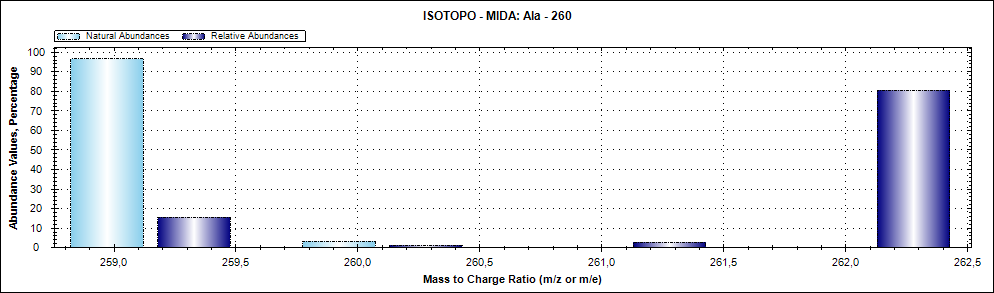


Figure 4a Ala 260 Spectrum Analysis


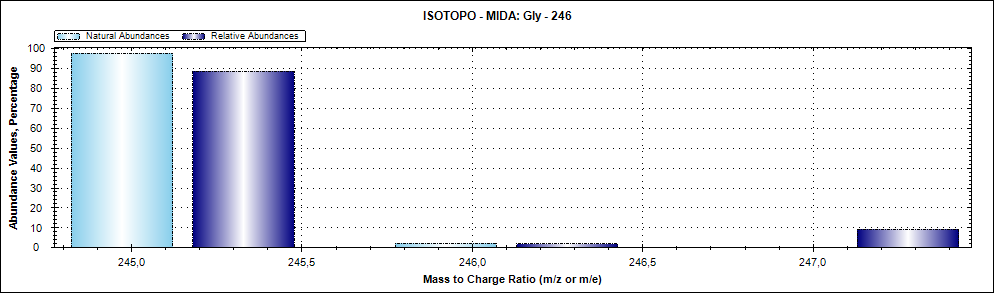


Figure 4b Gly 246 Spectrum Analysis


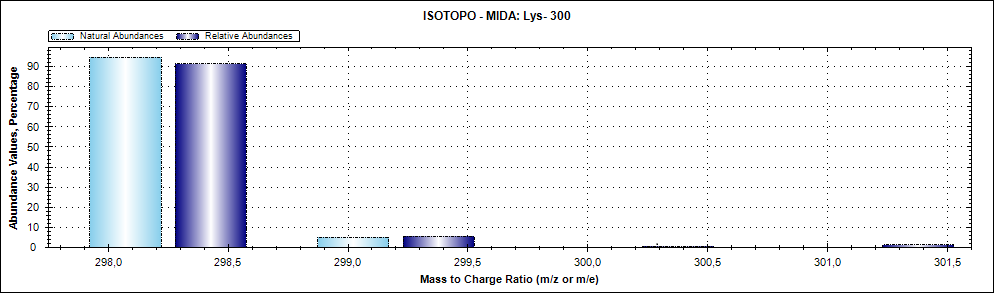


Figure 4c: Lys 300 Spectrum Analysis


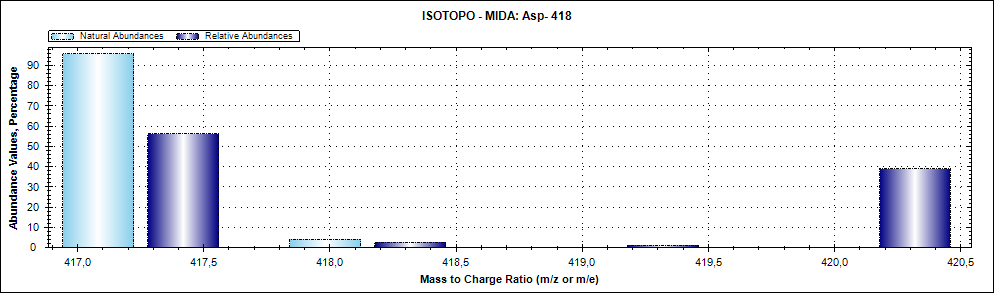


Figure 4d: Asp 418 Spectrum Analysis


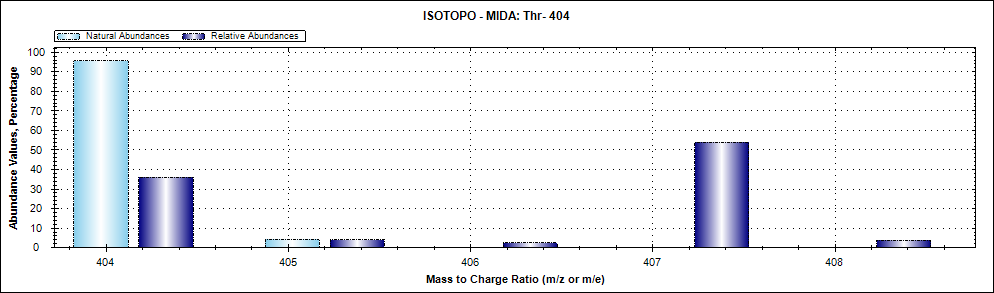


Figure 4e: Thr 404 Spectrum Analysis


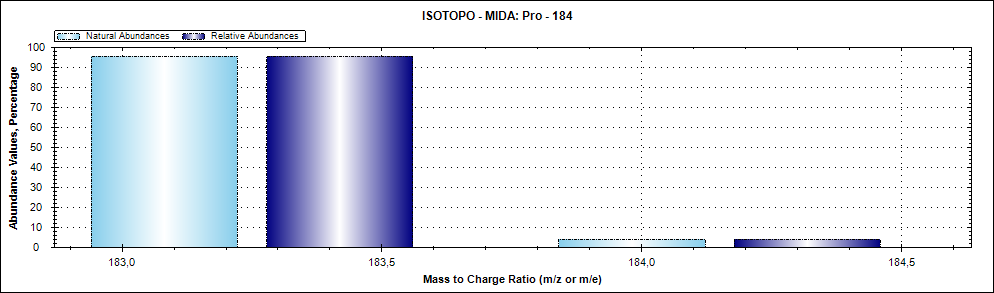


Figure 4f: Pro 184 Spectrum Analysis


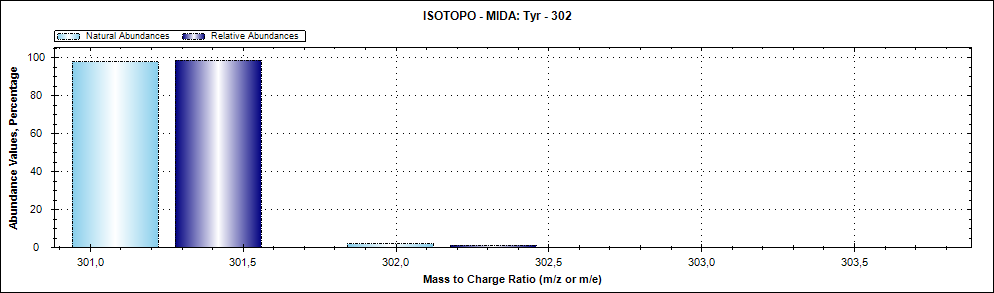


Figure 4e: Tyrosine 302 Spectrum Analysis

**Figure 4. Isotopo Spectrum Graphics**


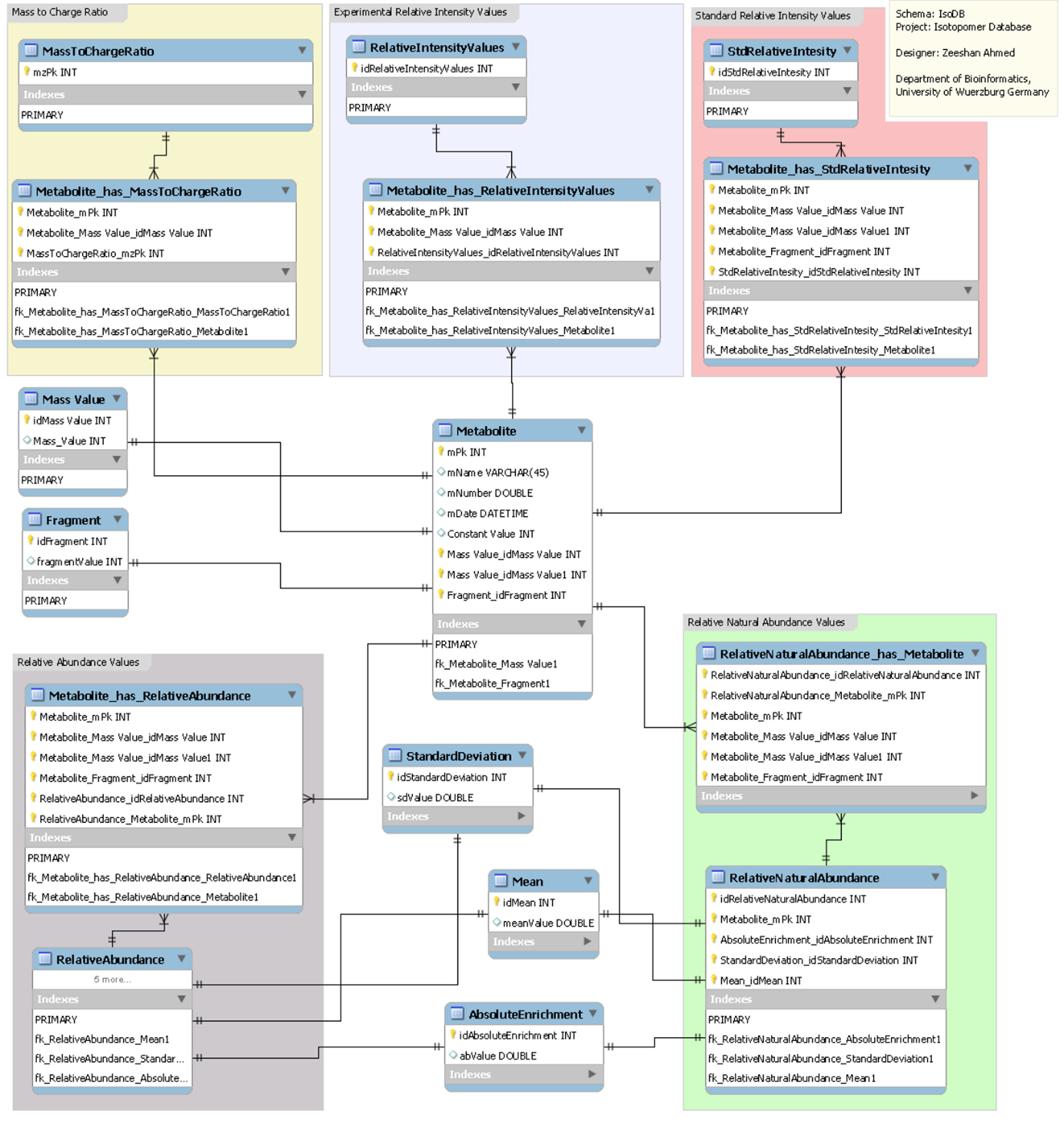


**Figure 5. Isotopo Database Schema**

This Figure contains the following relations: Metabolite, MassToChargeRatio, Metabolite_has_MassToChargeRatio, elativeIntensityValues,Metabolite_has_RelativeIntensityValues, StdRelativeIntesity,Metabolite_has_StdRelativeIntesity, Mass Value, FragmentMetabolite_ has_RelativeAbundance, RelativeAbundance, RelativeNaturalAbundance_has_Metabolite, RelativeNaturalAbundance, Standard Deviation, Mean, AbsoluteEnrichment.


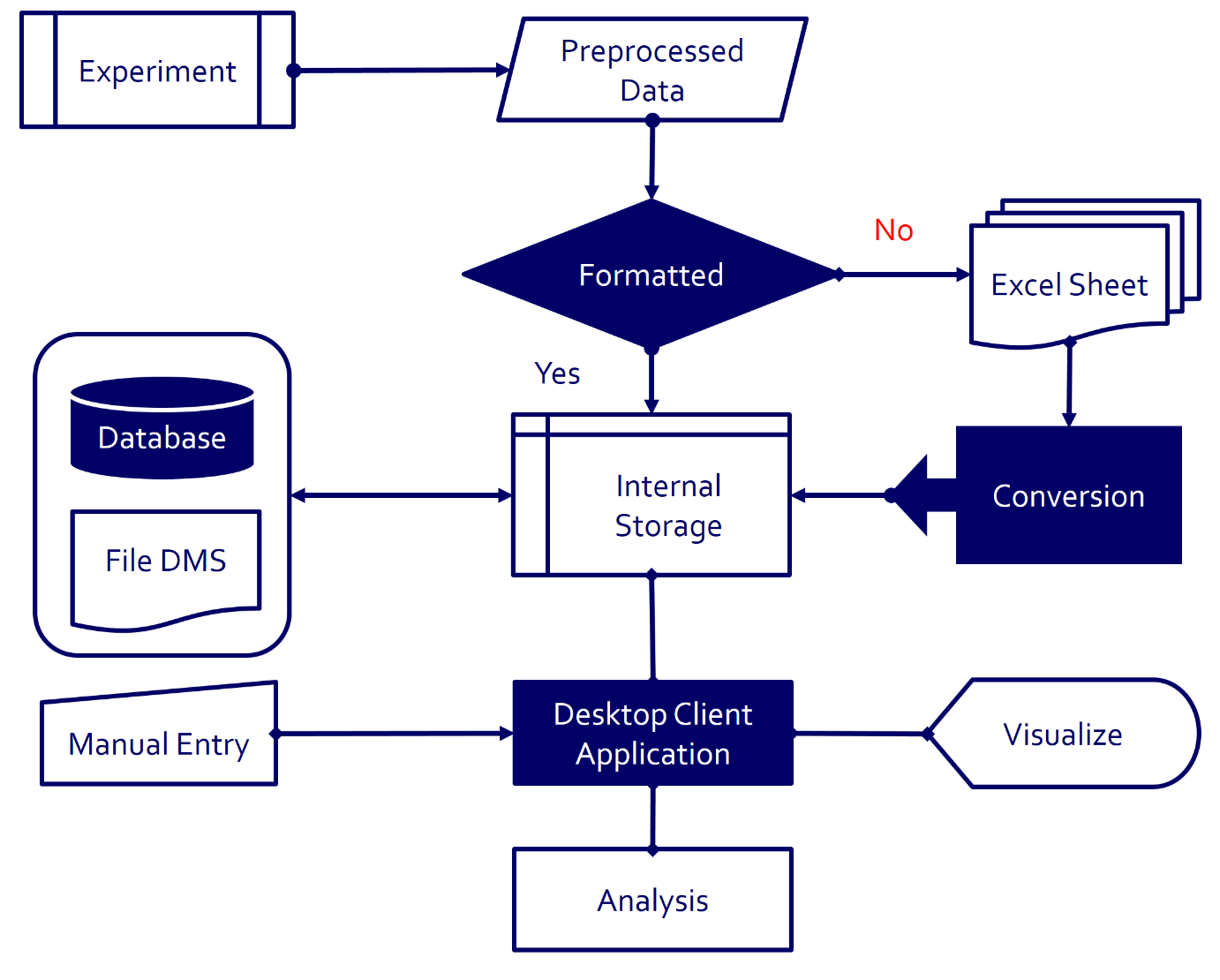


**Figure 6. Conceptual System Design of Isotopo**


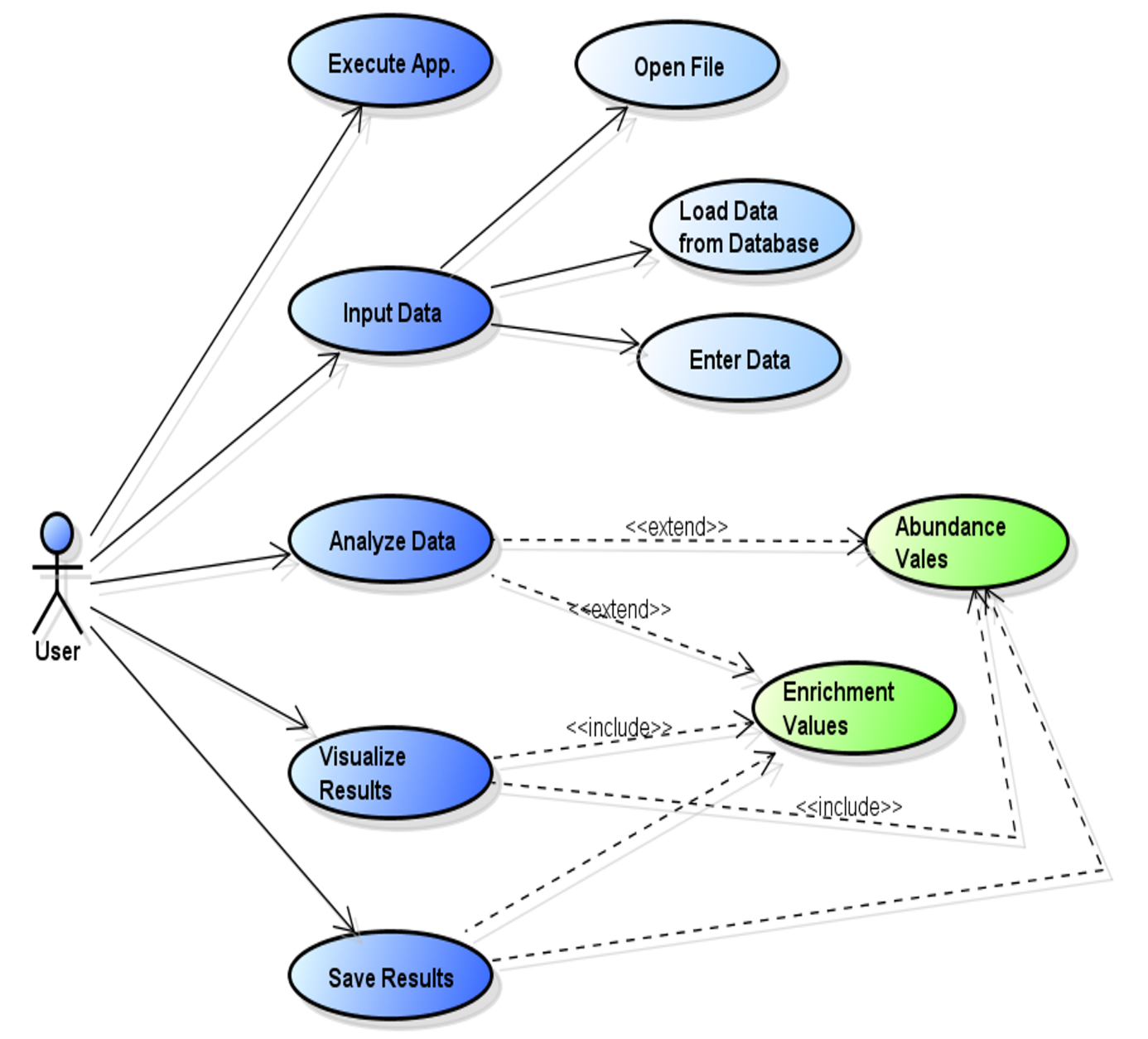


(**A**). The use case diagram of Isotopo is consisting of a User, five direct (Execute Application, Input Data, Analyze Data, Visualize Data and Save Results), three remote activities (Open File, Load Data from Database, Enter Data), two extended and included activities (abundance values and enrichment cvalues).


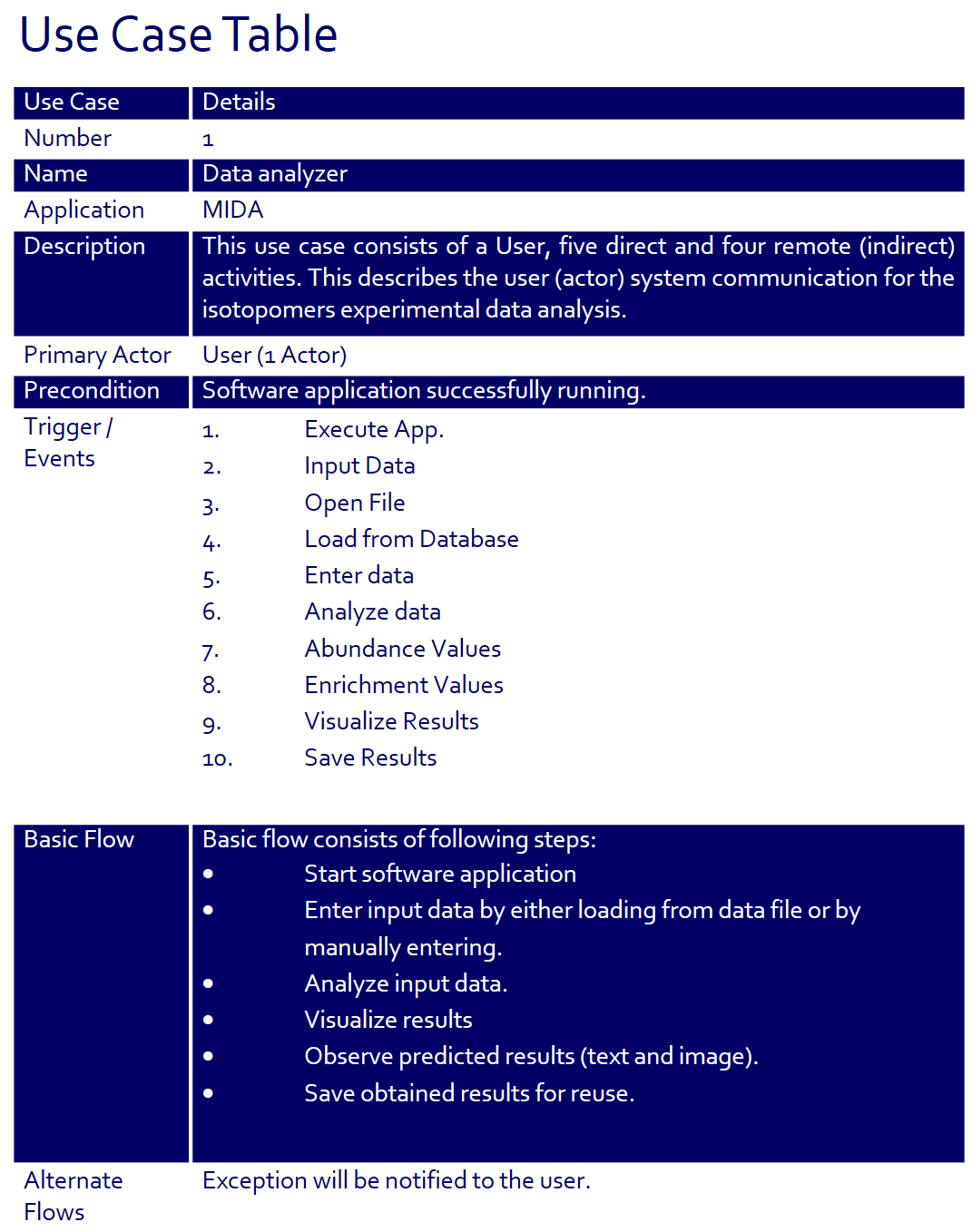


(**B**) Tabular details

**Figure 7. Use Case Design (A) and Table (B) of the Isotopo**


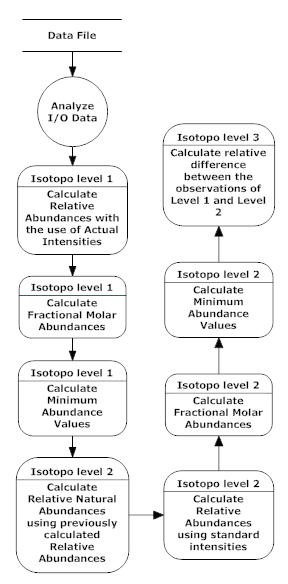


**Figure 8. Data Flow Diagram of Isotopo.** The data flow diagram of Isotopo is consisting of a File (Data File), one main Function (Analyze I/O Data) and eight internal functions: Calculate Natural Abundance Values and Calculate Relative Abundance Values, Calculate Fractional Molar Abundance, Calculate Minimum Abundance Values, Calculate Relative Natural Abundances using previously calculated Relative Abundances, Calculate relative difference between the observations of Level 1 and Level 2, Calculate new Minimum Abundance, Calculate new Fractional Molar Abundance and Calculate Relative Abundances using standard intensities.


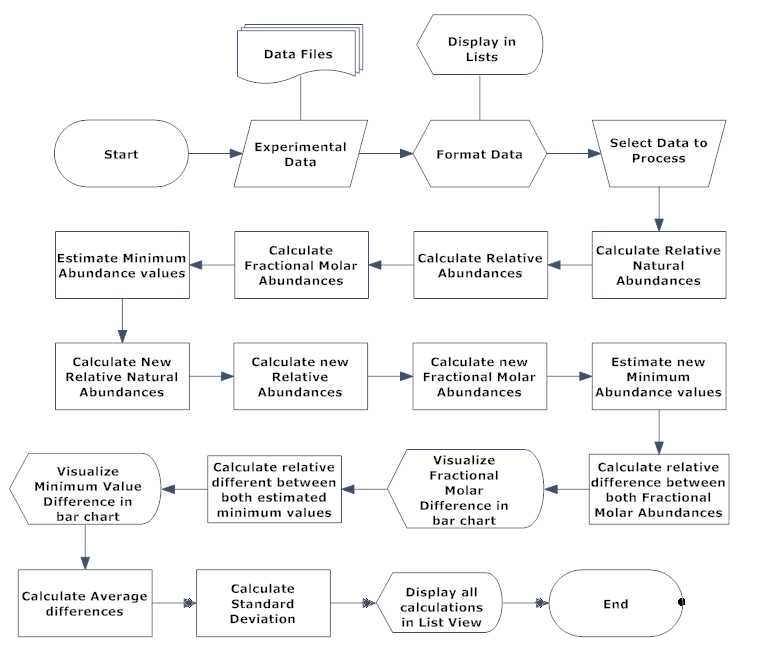


**Figure 9. Flow Chart of Isotopo.** Visual presentation of the UML based flow chart. The implemented flow of operations performed during experimental data input, processing, analysis and visualization is given. The flow chart of Isotopo Data Analyzer is consisting of one starting point (Start), one Input point (Experimental Data; Data Files), one formatting point (Format Data; Display in lists), one data selection point (Select Data to Process), processing units (Calculate Natural Abundance Values, Relative Abundance Values, Fractional Molar Abundance, Minimum Abundance Values, Average Differences and Standard Deviation), one visualization mode (Draw Spectrum) and one ending point (End).


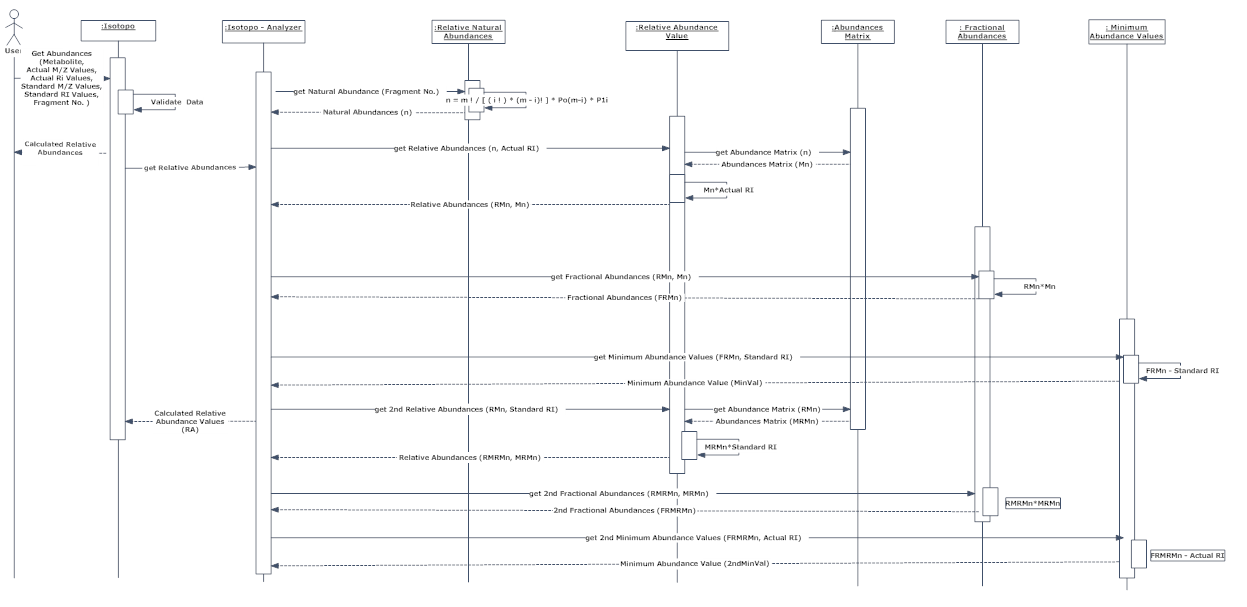


**Figure 10. System Sequence of Isotopo.** The system sequence diagram of Isotopo is consisting of repetitive iterative seven steps (Isotopo, Analyzer, Relative Natural Abundances, Relative Abundance Values, Abundance Matrix, Fractional Abundances and Minimum Abundance values) with several directing arrows in between.


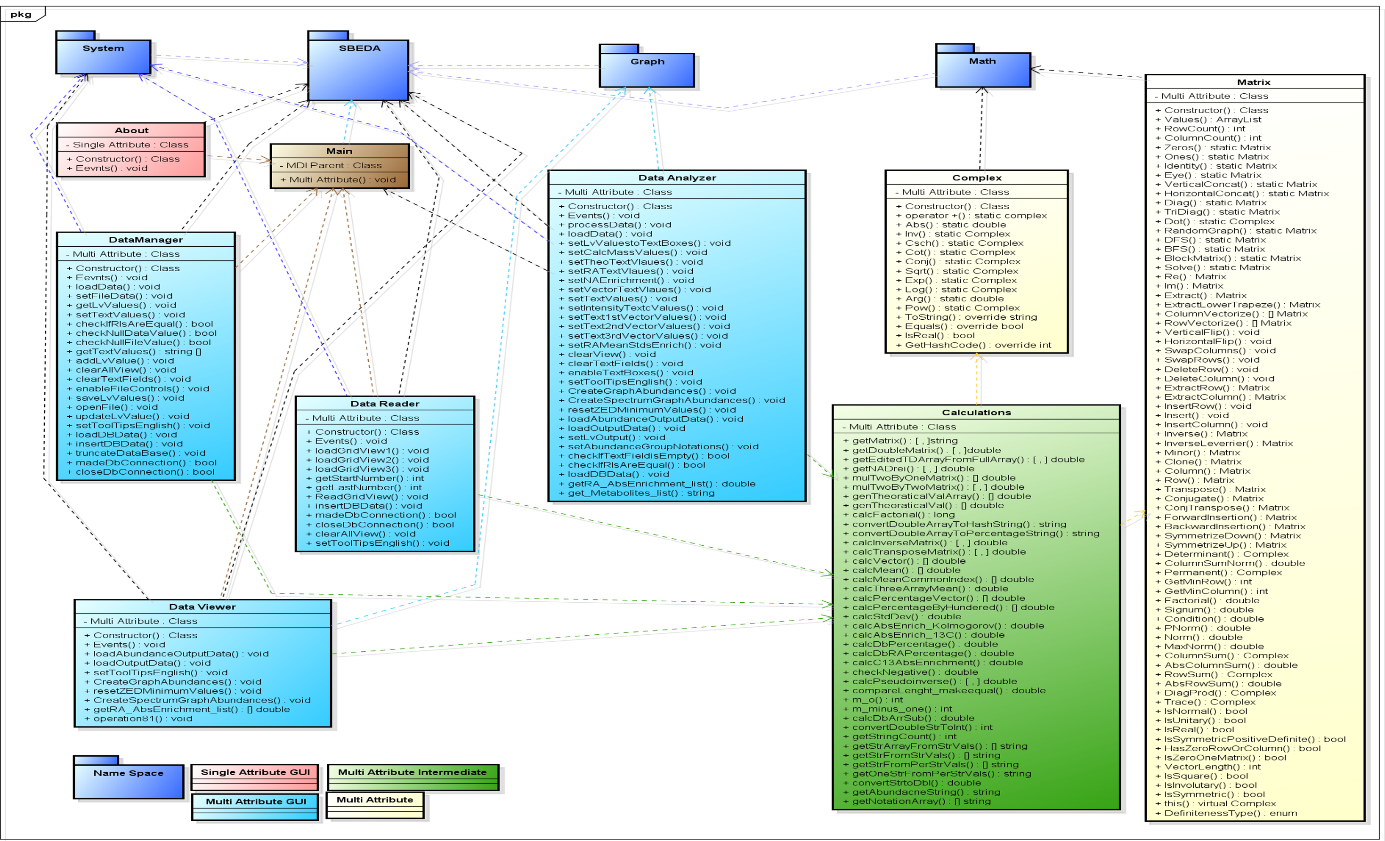


**Figure 11. Class Diagram of Isotopo.** The class diagram of Isotopo is consisting of four name spaces (System, SBEDA, Graph and Math), one main class (Main), four multi attribute graphical user interface classes (DataAnalyzer, DataManager, Data Reader and Data Viewer), two multi attribute classes (Complex and Matrix), one multi attribute intermediate class (calculations) and one single attribute graphical user interface class (About).


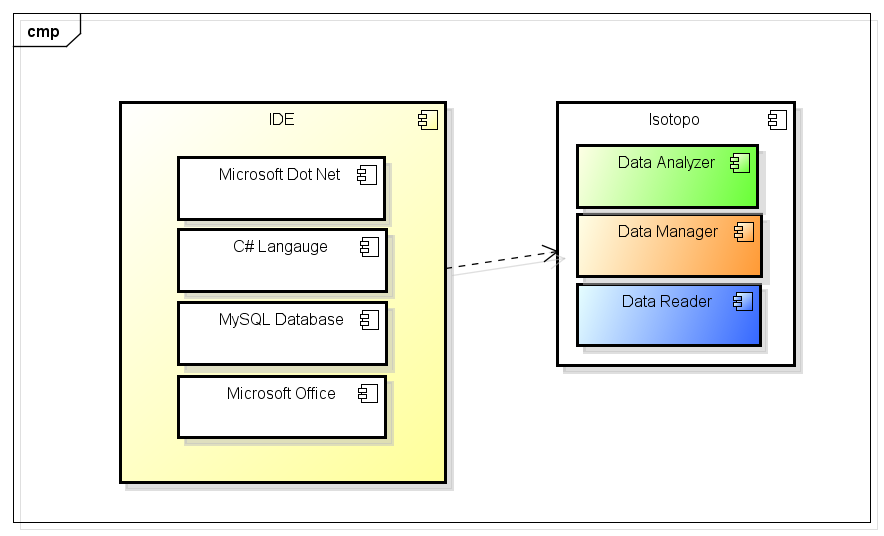


**Figure 12. Component Diagram of Isotopo.** The components diagram of the Isotopo is consisting of Integrated Development Environment (IDE) which includes following technologies: Microsoft Dot Net framework, C# programming language, MySQL Database and Microsoft Office (excel). Furthermore the diagram shows the components of the Isotopo application (Data Analyzer, Data Manager and Data Reader) and draws relationship with the IDE.
